# Predicting Clinical Management Decisions from Referral Information in Child and Adolescent Psychiatry Consultations: A Temporal Transportability Study

**DOI:** 10.3390/healthcare14213575

**Published:** 2026-10-22

**Authors:** Seda Güneysu, Ayşe Merve Benk Ayaz

**Affiliations:** Department of Child and Adolescent Psychiatry, Faculty of Medicine, Tokat Gaziosmanpaşa University, 60250 Tokat, Turkey; aysemerve.ayaz@gop.edu.tr

**Keywords:** child and adolescent psychiatry, consultation-liaison psychiatry, health informatics, clinical prediction model, machine learning, natural language processing, temporal transportability

## Abstract

**Highlights:**

**What are the main findings?**

- Predictive performance and temporal transportability differed markedly across the six management domains.
- Referral composition and management-outcome prevalence changed substantially between service periods.

**What are the implications of the main findings?**

- Internal model performance should not be assumed to persist when consultation pathways and service mix change.
- Referral-note text can contribute within a fitted model without demonstrating clear incremental temporal performance beyond structured referral information.

**Abstract:**

**Background/Objectives:** Child and adolescent psychiatry consultation-liaison services address heterogeneous management demands, while referral pathways and service organization may change over time. We examined whether information available before psychiatric assessment could predict six subsequent documented clinical management decisions and whether predictive performance remained transportable to a later service period. **Methods:** This retrospective prediction study included 656 unique patients aged 1–18 years. Six management domains coded from psychiatric consultation-response documentation were modeled using referral-note text, age, sex, and clinically harmonized referring department. L2-regularized logistic regression models incorporated term frequency–inverse document frequency text features. Internal performance was assessed using grouped repeated cross-validation. Temporal transportability was evaluated by applying models developed on 2013–2019 records (*n* = 447) to the 2022–2025 cohort (*n* = 209) without retraining or recalibration. The area under the precision–recall curve (AUPRC) was the primary metric. **Results:** Full-cohort cross-validated AUPRC ranged from 0.282 to 0.721. Temporal AUPRC ranged from 0.136 for psychosocial management to 0.663 for medication management decision; corresponding values were 0.282 for additional assessment, 0.586 for follow-up/reassessment, 0.415 for safety/disposition, and 0.575 for liaison/medical integration. Temporal discrimination was particularly weak for psychosocial management (AUROC = 0.532) and remained limited for additional assessment (AUROC = 0.587). Referring-department composition and several outcome prevalences changed substantially between periods. Although referral-note text contributed within the fitted models, its incremental temporal value over structured predictors was not established after false discovery rate correction; confidence intervals remained compatible with modest benefit or harm. **Conclusions:** Pre-assessment information contained management-domain-specific predictive signal for subsequent documented clinical management decisions, but transportability varied across clinical targets and service periods. These findings should be interpreted as evidence about temporal transportability rather than clinical implementation readiness. The results do not support automated clinical decision-making. Multicenter, preferably prospective, validation and evaluation of clinical utility and workflow impact are required before implementation.

## 1. Introduction

Child and adolescent psychiatry consultation-liaison services operate at the interface between mental health care and broader hospital-based services. Their role extends beyond diagnostic assessment and may involve medication management, psychosocial intervention, additional assessment, follow-up planning, safety and disposition decisions, and coordination with medical teams [1]. From a healthcare-delivery perspective, this creates a heterogeneous clinical workload rather than a single consultation task: patients referred through the same service may ultimately require substantially different forms of management. Recent systematic evidence has likewise shown considerable variation across pediatric mental health liaison services in patient characteristics, service organization, clinical outcomes, stakeholder experiences, and service-level effects [2]. Yet relatively little is known about how much information available at the point of referral anticipates the different management decisions made after psychiatric assessment, or whether such information remains informative when referral patterns and service conditions change over time.

Referral documentation is particularly relevant to this question because it is generated before specialist assessment and may contain clinically meaningful information that is not fully represented by routine structured variables. Referral notes can describe the reason for consultation, problems recognized by the referring clinician, expectations of the psychiatric assessment, and aspects of the surrounding clinical context. Natural language processing (NLP) provides a means of incorporating this unstructured information into prediction models alongside structured data. However, research using NLP in child and youth mental health has largely focused on identifying specific psychopathological states or risk conditions. In a systematic review of 12 studies, Ibrahim et al. [3] found that suicide risk, depression, and stress were among the most frequently examined targets. Clinical-record studies have similarly used free text to identify suicidal behavior in adolescents [4], while studies in broader healthcare settings have used early clinical documentation to predict emergency-department disposition [5] or subsequent mental health service use [6]. These applications demonstrate that clinically useful predictive information may be present in text generated early in the care pathway. Predicting a diagnosis, a single risk state, or later service use, however, is a different problem from examining whether referral-time information contains distinct predictive signals for the multiple documented management decisions that follow specialist psychiatric assessment.

A second issue is whether such predictive information remains transportable when the healthcare environment changes. Prediction performance is not determined by model architecture alone. Patient mix, referral sources, documentation practices, clinical workflows, and outcome prevalence may all change over time, altering both the information available to a model and the clinical decisions it is intended to predict. Temporal dataset shift can therefore affect model performance even within the same institution [7]. Contemporary validation frameworks similarly emphasize that internal evaluation does not establish generalizability and that prediction models should be examined across new time periods, locations, and clinical contexts [8]. This issue is particularly relevant to consultation services, in which changes in referral pathways or service organization may substantially alter the population seen by the clinical team. In adolescent suicide-attempt prediction, for example, Zang et al. [9] showed that transported model performance could either decrease or improve across real-world data sources and that more complex machine-learning approaches did not consistently outperform regularized logistic regression. Temporal evaluation should therefore be interpreted in relation to concurrent changes in service mix, case mix, and outcome prevalence rather than as a simple test of whether a model has deteriorated with time.

Against this background, the present study examined whether information available before child and adolescent psychiatry assessment could predict six distinct subsequent documented clinical management decisions and whether these predictive patterns remained transportable across different service periods. Predictors were deliberately restricted to referral-note text, age, sex, and clinically harmonized referring department. Information generated during the psychiatric consultation, including the consultation response from which the outcomes were derived, was excluded from the supervised models, as were diagnostic variables whose availability before assessment could not be reliably established. Internal performance was evaluated using grouped repeated cross-validation, and temporal transportability was assessed by applying models developed on 2013–2019 records to the 2022–2025 cohort without retraining or recalibration. We also examined whether referral-note text provided incremental predictive value beyond routinely available structured information. We expected performance to differ across management domains and temporal transportability to be heterogeneous. The study was framed as a temporal transportability analysis rather than as the development of a deployable clinical decision-support system. Its purpose was to determine how reliably routinely collected pre-assessment information predicts subsequent documented management decisions under changing real-world service conditions. Establishing the extent and limits of this transportability provides an empirical basis for future studies evaluating whether such information can contribute to consultation workflow and service planning.

## 2. Materials and Methods

### 2.1. Study Design, Setting, and Sample

**Figure 1:**
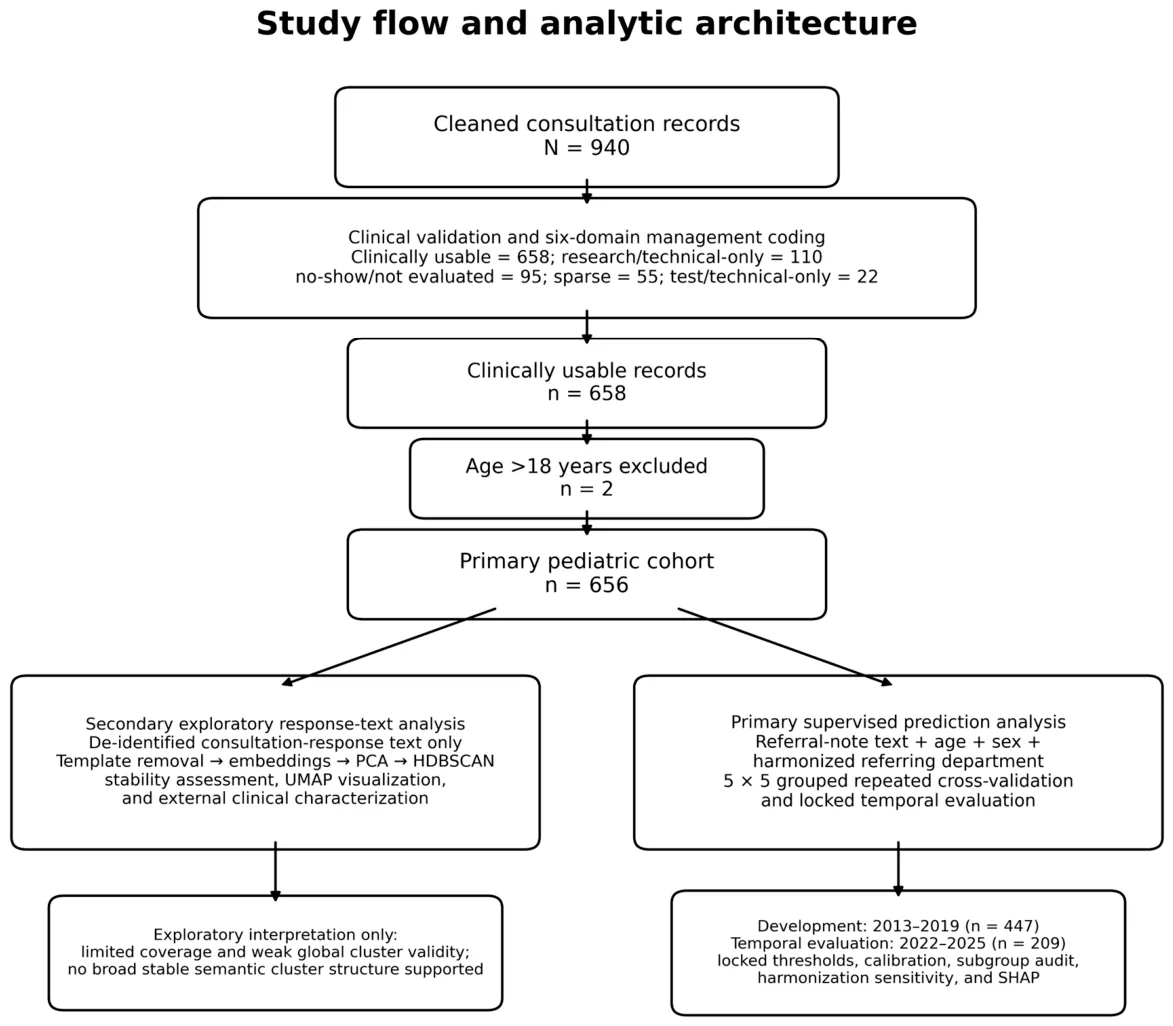
Study flow and analytic architecture. The primary supervised branch used referral-note text, age, sex, and the clinically harmonized referring department. The secondary exploratory branch used only de-identified consultation-response text and was conducted separately from the supervised prediction pipeline; it was interpreted as an analysis of semantic/documentation structure rather than clinical phenotype discovery. PCA = principal component analysis; HDBSCAN = Hierarchical Density-Based Spatial Clustering of Applications with Noise; UMAP = Uniform Manifold Approximation and Projection; SHAP = SHapley Additive exPlanations.

This single-center, retrospective clinical prediction modeling study examined whether information available at the time of referral for a child and adolescent psychiatry consultation could predict clinical management decisions documented after psychiatric assessment, and whether this predictive information remained transportable to a later service period. Reporting was structured in accordance with the TRIPOD+AI guidelines [10]. The study flow and overall analytic framework are illustrated in Figure 1.

Data were obtained from consultation records evaluated by the Department of Child and Adolescent Psychiatry at Tokat Gaziosmanpaşa University Faculty of Medicine. From an initial set of 940 cleaned records, we excluded 110 research- or technically oriented records, 95 involving patient non-attendance or non-evaluability, 55 with psychiatric responses insufficient for reliable clinical coding, and 22 containing only a test or technical procedure. Of the remaining 658 clinically usable records, two involving patients older than 18 years were excluded, yielding a primary cohort of 656 unique patients aged 1–18 years. Each patient was represented by a single consultation. Because there were no repeated consultations within patients, additional patient-level clustering adjustment was unnecessary. Clinician identifiers were not retained after de-identification; therefore, clinician-level clustering and changes in clinician composition across periods could not be assessed or modeled.

Records spanned 2013–2016, 2018–2019, and 2022–2025. The absence of records in 2017 reflected limited continuity among clinicians able to provide child and adolescent psychiatry consultations, whereas the 2020–2021 gap corresponded to the COVID-19 pandemic and associated administrative workflow changes. Consequently, the temporal analysis was conceptualized as an assessment of transportability across interrupted historical and service periods, rather than as a simple test of model aging. No probabilistic sampling scheme was employed; all records meeting eligibility criteria within the defined clinical archive were included. The primary cohort consisted of 447 patients in the 2013–2019 development period and 209 in the 2022–2025 temporal evaluation period. No a priori sample-size calculation was performed; the available sample was determined by all eligible records within the defined clinical archive. Because the area under the precision–recall curve (AUPRC) is sensitive to outcome prevalence, prevalence rates were reported separately for each period.

### 2.2. Outcome Variables, Reference Standard, and Reliability

Clinical management decisions were coded using a six-domain framework finalized prior to supervised modeling: medication management decision, psychosocial management, additional assessment, follow-up/reassessment, safety/disposition, and liaison/medical integration. These domains were intended to represent distinct management functions encountered in consultation-liaison practice, spanning treatment decisions, psychosocial intervention, further diagnostic or clinical assessment, follow-up planning, acute safety/disposition, and coordination with medical teams. Each domain was originally scored on an ordinal scale from 0 to 2. For the primary models, scores were dichotomized as ≥1 versus 0, indicating the presence of any documented management decision within that domain. The primary binary formulation therefore addressed whether a management action was documented, rather than the intensity of that action. Because this dichotomization combines lower- and higher-intensity decisions, the original ordinal ratings were retained and a separate sensitivity analysis examined score 2 versus scores 0–1. The medication management outcome was not restricted to the initiation of a new prescription; it also encompassed explicit decisions to continue, modify, conditionally initiate, or discontinue treatment, as well as decisions to monitor without medication. Accordingly, this outcome is referred to throughout as a medication management decision.

The original 0–2 ratings were assigned by a child and adolescent psychiatrist based solely on the psychiatric consultation-response text. Referral-note text was not used as evidence for outcome coding. Because the response text served as the source for outcome labels, it was excluded from all supervised predictor sets. The resulting reference standard should therefore be interpreted as a reproducible coding of documented management decisions rather than as an independent measure of clinical need. In particular, a score of 0 indicates that the corresponding management decision was not documented according to the coding framework; it does not establish that the underlying clinical need was absent. Diagnostic information was similarly excluded from the primary models, as its availability prior to assessment could not be reliably established for every case. During the final quality audit of the broader screened record set, 242 of 5400 domain-level ratings (4.5%) among the first 900 records were revised. An additional 66 documentation-to-evidence mapping corrections were made without altering the corresponding score. The coding framework, cohort derivation, outcome distributions, and reliability results are detailed in Supplementary Table S1.

To assess the reproducibility of the reference standard, 150 cases were selected by simple random sampling without replacement from the primary cohort using a prespecified random seed (20260827). A second child and adolescent psychiatrist reviewed only the consultation-response text and the frozen coding guide, blinded to the first rater’s scores, referral notes, model predictions, and prior results. The reliability exercise was designed to assess reproducibility of the documentation-based coding rules, rather than independent agreement on clinical need or treatment appropriateness. The frozen coding guide used explicit documentation criteria for each domain and score, and the reliability sample was randomly selected rather than chosen on the basis of documentation clarity. For the ordinal 0–2 ratings, exact agreement, linearly weighted Cohen’s κ, quadratically weighted κ (as a sensitivity measure), and Gwet’s AC2 were calculated. For the binary ≥1 versus 0 outcomes, percentage agreement, Cohen’s κ, and Gwet’s AC1 were estimated. Ninety-five percent confidence intervals (CIs) for reliability coefficients were derived from 5000 paired bootstrap resamples.

### 2.3. Predictors, Preprocessing, and Modeling

Primary models relied exclusively on information available prior to psychiatric assessment: referral-note text, age, sex, and clinically harmonized referring department. Consultation-response text, manually coded management scores, model-derived variables, and post-assessment information were excluded. No additional structured variables representing family or socioeconomic context, previous psychiatric care, caregiver capacity, or contemporaneous service availability were included in the supervised predictor set. There were no missing values for age, sex, referring department, referral-note text, or any of the six outcomes. Sex was recorded as male or female.

Administrative subdivisions and numbered outpatient units belonging to the same specialty were consolidated to mitigate institution-specific administrative signaling. Low-frequency units were grouped under clinically meaningful higher-level categories, while clinically distinct care settings—such as outpatient clinics, inpatient wards, intensive care units, and the emergency department—were preserved as separate entities where appropriate. This harmonization yielded 17 clinical categories. The original administrative department variable was retained for sensitivity analyses, but the harmonized variable was utilized in the primary models.

Referral-note text underwent Unicode NFKC normalization. Turkish uppercase I and İ were converted to ı and i, respectively, prior to lowercasing. Non-alphanumeric characters—excluding Latin letters and the Turkish characters ç, ğ, ı, ö, ş, and ü—were replaced with spaces, followed by the removal of repeated, leading, and trailing spaces. Text was represented via word-level 1–2-gram TF-IDF features (min_df = 3, max_df = 0.98, max_features = 12,000, sublinear term frequency, L2 normalization). The supervised text representation was therefore based on sparse lexical features; contextual embeddings, transformer-derived representations, and dedicated negation modeling were not incorporated into the primary prediction models. Age was standardized, while sex and the harmonized referring department were one-hot encoded. All learnable preprocessing steps, including the TF-IDF vocabulary and inverse document frequencies, were fitted exclusively on the corresponding training data.

Exactly identical normalized referral notes were mapped to a common SHA-1-based group identifier. This guaranteed that the same referral-note group could not appear in both the training and held-out evaluation partitions during grouped cross-validation.

The six management outcomes were modeled independently. Each model incorporated TF-IDF features, age, sex, and the harmonized referring department. The classifier was L2-regularized logistic regression (C = 1.0, liblinear solver, maximum 4000 iterations) without explicit interaction terms. No class weighting, oversampling, or undersampling was used in the primary models. No supervised comparison with contextual language representations was performed. Neither the model architecture nor its hyperparameters were reselected using temporal evaluation data. Final model specifications and target-leakage checks are summarized in Supplementary Table S2.

### 2.4. Full-Cohort Internal Cross-Validation and Temporal Evaluation

Full-cohort internal performance was assessed across all 656 patients using 5-fold × 5-repeat StratifiedGroupKFold cross-validation. Identical normalized referral-note groups were retained within the same fold. TF-IDF and all other learnable transformations were refit using training data only within each fold. Predictions were generated solely for held-out cases, and repeated out-of-fold probabilities for each patient were averaged across repetitions.

The primary performance measure was AUPRC (average precision). The area under the receiver operating characteristic curve (AUROC), Brier score, log loss, calibration intercept, and calibration slope were examined as supporting measures [11]. Ninety-five percent CIs for AUPRC and AUROC were obtained from the 2.5th and 97.5th percentiles of 2000 nonparametric bootstrap resamples at the patient level. Because AUPRC depends on outcome prevalence, its interpretation was considered alongside the corresponding period-specific prevalence rather than in isolation.

Temporal transportability was evaluated separately. Models were developed using only 2013–2019 records (*n* = 447) and applied to the 2022–2025 cohort (*n* = 209) without retraining, recalibration, threshold optimization, or hyperparameter tuning. No intercept or slope recalibration was performed because the purpose of this analysis was to evaluate unadapted temporal transportability of the development-period models. The later-period cohort was treated not as a conventional, randomly held-out sample from the same temporal distribution, but rather as a subsequent clinical cohort observed under a distinct service composition. Differences in referring-department distributions across periods were assessed using the chi-square test and Cramér’s V. Period-specific prevalence and absolute prevalence differences were also examined for each management outcome. Internal cross-validation and temporal evaluation were therefore used to address different questions: the former estimated performance under internal resampling, whereas the latter examined whether the fitted prediction strategy remained informative in a later service period. Full-cohort cross-validated and temporal estimates were interpreted as complementary evaluations rather than as directly comparable independent test samples, and differences between them were considered in relation to historical period, outcome prevalence, and service composition.

To examine the incremental value of referral-note text, two models were trained using the same 2013–2019 development data: a structured model containing age, sex, and harmonized referring department, and a combined model that additionally included TF-IDF referral-note features. Both were evaluated on the same 2022–2025 cases without retraining or recalibration, with all other model specifications held constant. ΔAUPRC was calculated as the combined model minus the structured model. Ninety-five percent CIs and two-sided *p* values were obtained from 2000 paired bootstrap resamples, and Benjamini–Hochberg false discovery rate (BH-FDR) correction was applied across the six outcomes. This paired temporal comparison was used to assess incremental predictive value of referral-note text; SHAP attribution was treated separately as a measure of within-model predictive contribution. Temporal evaluation data were not used for model training, hyperparameter selection, or threshold selection.

Decision thresholds were derived exclusively from grouped out-of-fold predictions within the 2013–2019 development period. Three operating points were considered: 0.50, the threshold maximizing Youden’s J statistic, and the threshold achieving at least 80% sensitivity. These thresholds were subsequently locked and applied unchanged to the temporal cohort. Sensitivity, specificity, positive predictive value, negative predictive value, F1 score, and balanced accuracy were calculated. These analyses were intended to characterize model behavior under different operating conditions rather than to define recommended clinical thresholds.

### 2.5. Sensitivity, Calibration, and Exploratory Subgroup Analyses

The effect of referring-department harmonization was evaluated by comparing models using the raw and harmonized department variables on identical training folds and held-out cases. Full-cohort internal comparisons utilized the same 5-fold × 5-repeat grouped cross-validation framework, whereas temporal comparisons employed the same 2013–2019 to 2022–2025 split. Paired differences between harmonized and raw models were calculated for AUPRC, AUROC, and Brier score. Ninety-five percent CIs and two-sided *p* values were obtained from 2000 paired bootstrap resamples, with BH-FDR correction applied within each evaluation setting and metric family (Supplementary Table S3, Panel A; Supplementary Figure S3A–C).

Sensitivity to regularization strength was examined by repeating the workflow with C = 0.1, 0.3, 1, 3, and 10. This analysis was not intended to select a new optimal value of C; the prespecified primary value of C = 1.0 was retained, and the analysis served only to assess whether the overall performance pattern was robust to regularization strength (Supplementary Table S3, Panel B). In a separate archived supporting analysis, score 2 was modeled against scores 0–1 for each domain to examine the sensitivity of the primary ≥1 versus 0 definition to higher-intensity management decisions. This analysis specifically assessed whether the main performance pattern depended on combining lower- and higher-intensity documented management decisions within the primary binary outcome definition. It did not alter the primary outcome definition or model selection (Supplementary Table S3, Panel C).

Temporal calibration was assessed using the Brier score, calibration intercept, calibration slope, and graphical calibration curves [12] (Supplementary Figure S2A–F). These analyses evaluated the calibration of the unchanged development-period models in the later cohort; no temporal recalibration was applied. Exploratory supplementary decision-curve analyses were conducted for psychosocial management and additional assessment across threshold probabilities from 0.01 to 0.50, comparing model net benefit with manage-all and manage-none strategies [13]. The decision-curve analyses were used only to characterize possible threshold-dependent net benefit and were not interpreted as evidence of established clinical utility, recommended thresholds, or implementation readiness (Supplementary Figure S2G,H).

Performance heterogeneity within the temporal cohort was explored by sex (male/female) and developmental age group (1–11/12–18 years). For each subgroup and outcome, sample size, event count, prevalence, AUPRC, AUROC, Brier score, and sensitivity and specificity at the locked Youden threshold were reported. CIs for AUPRC and AUROC were obtained using 2000 bootstrap resamples. These analyses were post hoc and descriptive, and estimates based on small event counts were interpreted cautiously. They were not treated as formal tests of algorithmic fairness or as evidence of the absence of bias (Supplementary Table S4, Panel C; Supplementary Figure S3D,E).

As an additional sensitivity analysis, reliability of the reference standard was re-examined separately within the 2013–2019 and 2022–2025 periods using the same blinded 150-case sample (92 and 58 cases, respectively). Percentage agreement, Cohen’s κ, and Gwet’s AC1 were reported for the binary outcomes, and exact agreement, linearly weighted κ, and Gwet’s AC2 for the ordinal scores; no formal test of equality between periods was performed (Supplementary Table S1, Panel E). Because the harmonized referring department carried a substantial SHAP contribution for safety/disposition (Section 3.7), the association between harmonized department and safety/disposition prevalence was also examined descriptively in the full cohort and separately by period, with Wilson 95% CIs for department-specific prevalence. Because expected cell counts were sparse, a fixed-margin Monte Carlo conditional test (50,000 replicates) and bias-corrected Cramér’s V were used in preference to the asymptotic chi-square test (Supplementary Table S4, Panel D). The construction of the 17 harmonized referring-department categories and the rationale for each grouping are summarized in Supplementary Table S2, Panel C; raw administrative labels are not reproduced to protect institutional and personnel privacy.

### 2.6. Model Explainability and Secondary Exploratory Documentation Analysis

Global explainability of the final harmonized models was examined using Shapley additive explanations (SHAP) [14]. For each outcome, the model was refitted using the full *n* = 656 cohort, applying shap.LinearExplainer with shap.maskers.Independent and up to 200 background observations. Predictors were grouped into four prespecified blocks: referral-note text, harmonized referring department, age, and sex. For each block, mean absolute SHAP contributions were summed and expressed as a proportion of the total mean absolute contribution (Supplementary Table S5, Panel A). Because the models contained no explicit interaction terms, SHAP interaction analyses were not performed. SHAP values were interpreted only as measures of predictive contribution within the fitted models. They were not used to infer causal or treatment effects, clinical decision rules, or incremental predictive value beyond the other predictor blocks.

As a secondary exploratory documentation analysis, de-identified psychiatric consultation-response texts were examined independently of the supervised prediction pipeline. Following removal of standard documentation templates, text was represented using multilingual BGE-M3 embeddings [15], reduced to 50 principal components using principal component analysis (PCA), and clustered with Hierarchical Density-Based Spatial Clustering of Applications with Noise (HDBSCAN; min_cluster_size = 15, min_samples = 5) [16]. Uniform Manifold Approximation and Projection (UMAP) was used only for visualization and was not included as a clustering input [17]. The six manually coded management outcomes were not used for embedding generation, dimensionality reduction, clustering, or cluster selection. The use of contextual embeddings in this exploratory branch was separate from the primary supervised TF-IDF models and did not constitute a supervised comparison of text-representation approaches.

Robustness was examined through repeated subsampling, comparison of full-text and template-stripped solutions, and removal of duplicate or highly similar records. This exploratory branch did not influence outcome definitions, predictor selection, model fitting, or optimization of the supervised analyses. Detailed robustness results are provided in Supplementary Table S5, Panels B–D, and Supplementary Figure S1.

Observations labeled as “Noise” by HDBSCAN were compared with observations assigned to clusters to examine whether cluster assignment was associated with demographic, temporal, referral-source, documentation-length, or management characteristics. The “Noise” designation referred only to insufficient density for cluster assignment within the embedding space and did not indicate clinical non-classifiability. Continuous variables were compared using the Mann–Whitney U test with rank-biserial effect sizes, whereas categorical variables were analyzed using chi-square or Fisher’s exact tests as appropriate. *p* values for comparisons across the six management outcomes were corrected using BH-FDR. All resulting clusters were interpreted strictly as exploratory semantic or documentation patterns and not as validated clinical phenotypes.

### 2.7. Statistical Principles, Missing Data, Software, and Data Governance

Results were interpreted using point estimates, 95% CIs, prevalence, temporal transportability, and calibration, rather than *p* values alone. Statistical significance was therefore considered alongside effect magnitude and estimation uncertainty, particularly for temporal and incremental-value analyses. BH-FDR correction was applied to paired harmonization comparisons and prespecified multiple-outcome families. All hypothesis tests were two-sided with α = 0.05. Exploratory subgroup and unsupervised analyses were kept analytically distinct from the primary model-performance analyses and were not interpreted as confirmatory findings.

There were no missing values for age, sex, referring department, referral-note text, or any of the six outcomes in the primary cohort. Consequently, no exclusions due to missing outcomes or categorical missing-value classes were required. A median-imputation step for age remained technically present in the analytic pipeline; however, because age was complete, it had no effect on the results. No imputation parameters were learned from the temporal evaluation cohort.

Analyses were conducted in Python. Final supervised, sensitivity, and explainability analyses used Python 3.13.15, NumPy 2.1.3, pandas 2.2.3, SciPy 1.16.3, scikit-learn 1.6.1, statsmodels 0.15.0, and SHAP 0.52.0. Exploratory embedding and clustering analyses additionally utilized PyTorch 2.11.0+cpu, sentence-transformers 5.7.0, umap-learn 0.5.12, and stopwordsiso 0.7.1. HDBSCAN clustering used the hdbscan package (version 0.8.44). A random seed of 20260827 was fixed for supervised modeling and robustness analyses, and intermediate outputs and model files were checkpointed using data fingerprints and analysis signatures.

All direct personal identifiers were removed before analysis, and free-text clinical records were de-identified before computational processing. The study was not prospectively registered in a public registry. OpenAI ChatGPT (GPT-5.6 Sol) was used only for English-language translation and language editing during manuscript preparation; it was not used for study design, data collection, statistical analysis, or interpretation. All AI-assisted output was reviewed and verified by the authors.

## 3. Results

### 3.1. Sample, Outcome Distributions, and Reliability of the Reference Standard

Of the 940 consultation records initially identified, 658 contained sufficient information for clinical coding. After excluding two patients older than 18 years, the primary cohort comprised 656 unique patients aged 1–18 years (Figure 1; Supplementary Table S1). Of these, 447 belonged to the 2013–2019 development period and 209 to the 2022–2025 temporal evaluation period. There were no missing data among the primary predictors or outcomes. Across the full cohort, follow-up/reassessment was the most frequent management outcome (61.3%), followed by medication management decision (47.1%); prevalence across the remaining four domains ranged from 15.1% to 22.3%.

Reproducibility of the documentation-based reference standard was high in the independently and blindly reassessed sample of 150 consultations. Exact agreement for the ordinal ratings was 92.7% for medication management decision, 97.3% for psychosocial management, and 100% for each of the remaining four domains. The corresponding linearly weighted κ values were 0.892, 0.919, and 1.000, respectively. For the primary binary definitions, Cohen’s κ ranged from 0.893 to 1.000, with Gwet coefficients showing a similar pattern of agreement (Supplementary Table S1). These estimates reflect reproducibility of the prespecified documentation-coding rules rather than agreement on independent clinical need or treatment appropriateness.

### 3.2. Changes in Service Composition and Outcome Prevalence Across Periods

The clinical context differed substantially between the development and temporal evaluation periods. Referring-department distributions shifted markedly (χ^2^(16) = 159.62, *p* < 0.001; Cramér’s V = 0.493; Supplementary Table S4, Panel A). The largest change involved the anesthesiology outpatient clinic, which increased from 0.4% of referrals during the development period to 17.7% in the temporal cohort. Pediatric outpatient referrals decreased from 34.7% to 22.0%, and pediatric intensive care referrals decreased from 16.8% to 8.1%. The anesthesiology/maxillofacial surgery category, which was absent in the development period, accounted for 4.8% of the temporal cohort.

Outcome prevalence also changed in a domain-specific manner. Psychosocial management decreased from 24.6% to 10.5% (−14.1 percentage points; q < 0.001), and follow-up/reassessment decreased from 68.5% to 45.9% (−22.5 percentage points; q < 0.001). In contrast, liaison/medical integration increased from 18.1% to 31.1% (+13.0 percentage points; q < 0.001). Temporal differences in the other three management domains were not statistically significant after FDR correction (Supplementary Table S4, Panel B). Thus, the temporal cohort represented a materially different service composition rather than a later sample drawn from an otherwise stable clinical setting.

### 3.3. Full-Cohort Internal Performance and Temporal Transportability

**Figure 2:**
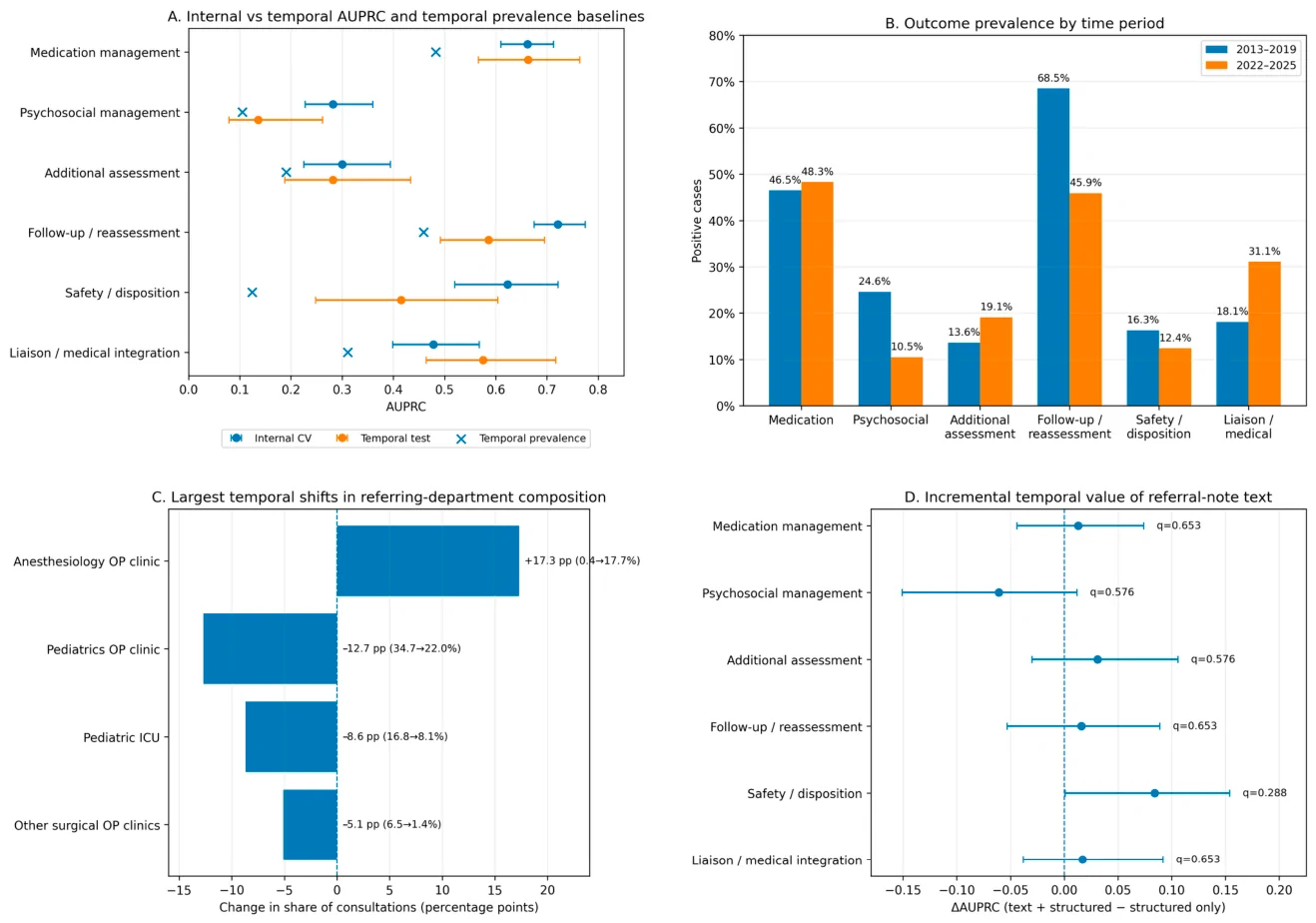
Temporal transportability, dataset shift, and incremental value of referral-note text. (**A**) Full-cohort internal cross-validated AUPRC and temporal AUPRC estimates for the six management domains, with 95% confidence intervals; temporal outcome prevalence is shown as the corresponding precision–recall baseline. (**B**) Outcome prevalence in the 2013–2019 development period and the 2022–2025 temporal evaluation period. (**C**) Largest changes in referring-department composition between the two periods, expressed as percentage-point differences in the share of consultations. (**D**) Paired change in temporal AUPRC after adding referral-note text to the structured predictors. Error bars represent 95% confidence intervals from 2000 paired bootstrap resamples; q values are Benjamini–Hochberg false discovery rate-adjusted across the six management domains. Positive ΔAUPRC values favor the combined text-plus-structured model. AUPRC = area under the precision–recall curve; CI = confidence interval; CV = cross-validation. In panel (**C**), arrows indicate the change from the 2013–2019 development period to the 2022–2025 temporal evaluation period.

Performance of the final harmonized models differed substantially across management domains (Table 1; Figure 2A). In full-cohort internal cross-validation, AUPRC ranged from 0.282 to 0.721. Follow-up/reassessment had the highest AUPRC (0.721; 95% CI, 0.675–0.775), whereas safety/disposition had the highest AUROC (0.901; 95% CI, 0.871–0.930).

Temporal performance remained heterogeneous. Medication management decision showed a temporal AUPRC of 0.663, close to the full-cohort cross-validated estimate of 0.662, although temporal AUROC was 0.664. Liaison/medical integration yielded an AUPRC of 0.575 and AUROC of 0.774, while safety/disposition yielded an AUPRC of 0.415 and AUROC of 0.781. For follow-up/reassessment, temporal AUPRC was 0.586 and AUROC was 0.649. In contrast, psychosocial management showed weak temporal discrimination, with an AUPRC of 0.136 (95% CI, 0.079–0.262) against a prevalence of 10.5% and an AUROC of 0.532 (95% CI, 0.396–0.665). Additional assessment also showed limited temporal discrimination (AUPRC = 0.282; AUROC = 0.587).

The temporal results therefore did not indicate uniform transportability across the six targets. Some domains retained appreciable discrimination in the later period, whereas others—most clearly psychosocial management and, to a lesser extent, additional assessment—did not.

### 3.4. Incremental Predictive Value of Referral-Note Text and Locked Threshold Performance

The change in temporal AUPRC after adding referral-note text to the structured predictors ranged from −0.061 to +0.084. None of the six management domains showed a statistically significant improvement after correction for multiple comparisons (all q ≥ 0.288; Figure 2D; Supplementary Table S3, Panel D). The largest positive difference was observed for safety/disposition (ΔAUPRC = +0.084; 95% CI, +0.001 to +0.154), although this difference did not remain statistically significant after FDR correction (q = 0.288). Confidence intervals were relatively wide for several domains and remained compatible with modest incremental benefit or harm. Accordingly, the present analysis did not establish incremental temporal value of referral-note text beyond the structured predictors; however, the available estimates were not sufficiently precise to conclude that such incremental value was absent.

At the Youden thresholds—derived exclusively during model development and locked before temporal evaluation—balanced accuracy ranged from 0.527 to 0.731 (Table 2). Safety/disposition had the highest balanced accuracy (0.731), with sensitivity of 0.577, specificity of 0.885, and positive predictive value of 0.417. Liaison/medical integration showed sensitivity of 0.800 and balanced accuracy of 0.671. Medication management decision yielded balanced accuracy of 0.570 despite its relatively stable cross-validated and temporal AUPRC estimates. The remaining operating characteristics are detailed in Table 2. These operating points were descriptive and should not be interpreted as clinically recommended thresholds.

### 3.5. Robustness and Sensitivity Analyses

Paired comparisons of raw and harmonized referring-department coding showed that harmonization did not produce uniform performance improvement (Supplementary Table S3, Panel A; Supplementary Figure S3A–C). During temporal evaluation, AUROC increased by 0.019 for safety/disposition (q = 0.009) and by 0.042 for liaison/medical integration (q < 0.001). For liaison/medical integration, the Brier score decreased from 0.187 to 0.175 (Δ = −0.012; q = 0.033). Conversely, harmonization was associated with a lower AUPRC for additional assessment (Δ = −0.039; q = 0.018) and a higher Brier score (Δ = +0.003; q = 0.024).

Varying the L2 regularization parameter across C = 0.1–10 did not materially alter the overall performance pattern, and no alternative value showed consistent superiority across outcomes and evaluation settings. Heterogeneity also persisted in the supporting analysis that defined higher-intensity management as score 2 versus scores 0–1. In this analysis, AUROC was 0.923 for safety/disposition, whereas psychosocial management yielded an AUPRC of 0.071 and an AUROC of 0.526 (Supplementary Table S3, Panels B and C).

Period-stratified reference-standard reliability remained high in both periods within the sensitivity sample. For medication management decision, Cohen’s κ was 0.891 in 2013–2019 and 0.897 in 2022–2025; for psychosocial management, κ was 0.912 and 0.937, respectively. The remaining four domains showed complete binary agreement within both periods, with a similar pattern for the ordinal ratings. Thus, the weak temporal performance observed for psychosocial management did not coincide with lower coding agreement in the later-period reliability subsample.

Safety/disposition prevalence varied markedly across the 17 harmonized referring-department categories, ranging from 0.5% (1/201) in the pediatrics outpatient clinic to 54.3% (50/92) in the pediatric intensive care unit. The association was significant in the full cohort (χ^2^(16) = 183.73; Monte Carlo *p* < 0.001; bias-corrected Cramér’s V = 0.506) and remained present within the 2013–2019 and 2022–2025 periods separately (bias-corrected V = 0.565 and 0.366, respectively; Supplementary Table S4, Panel D).

### 3.6. Calibration, Exploratory Decision Curves, and Subgroup Performance

Temporal calibration varied across management domains (Supplementary Figure S2A–F), indicating that calibration was not uniformly preserved when the development-period models were applied unchanged to the later cohort. For psychosocial management, weak discrimination was accompanied by an absence of sustained net benefit over both reference strategies across a broad and stable threshold range. The additional assessment model showed a more favorable net-benefit pattern at intermediate threshold probabilities, although this finding remained exploratory (Supplementary Figure S2G,H). These analyses were not used to define actionable thresholds or to infer readiness for clinical implementation.

Exploratory subgroup analyses suggested heterogeneity in temporal performance (Supplementary Table S4, Panel C; Supplementary Figure S3D,E). Temporal AUPRC for medication management decision was 0.770 in males and 0.465 in females; corresponding values for liaison/medical integration were 0.648 and 0.331. Several estimates, however, were based on small event counts. For safety/disposition, only eight positive events occurred among males and five among patients aged 1–11 years, resulting in substantial uncertainty. These subgroup findings were therefore treated as descriptive signals of possible performance heterogeneity rather than as formal evidence of between-group differences, algorithmic fairness, or absence of bias.

### 3.7. Model Explainability

**Figure 3:**
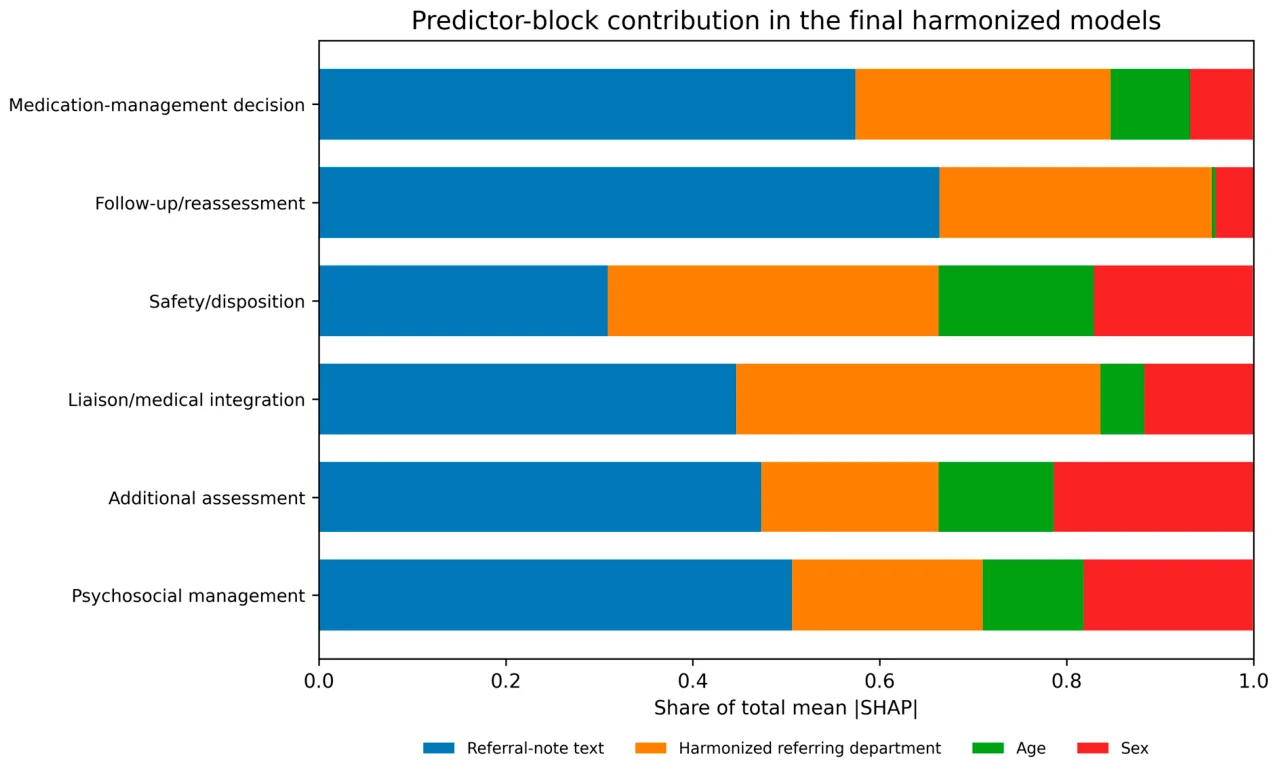
Predictor-block contribution in the final harmonized models. Stacked bars show the distribution of total mean absolute SHAP contribution across referral-note text, harmonized referring department, age, and sex. SHAP values quantify predictive contribution within the fitted model and should not be interpreted as causal effects or evidence of independent incremental value. SHAP = SHapley Additive exPlanations.

In the SHAP analysis, referral-note text accounted for the largest share of mean absolute predictive contribution in five of the six management domains (Figure 3; Supplementary Table S5, Panel A). Across these five outcomes, referral-note text represented 44.6–66.4% of the total mean absolute SHAP contribution, with the highest proportion observed for follow-up/reassessment (66.4%). For safety/disposition, harmonized referring department contributed the largest share (35.4%), followed by referral-note text (30.9%); age and sex generally contributed smaller proportions.

These findings describe how the fitted models distributed predictive contribution across available predictor blocks. They should not be interpreted as evidence that referral-note text provided independent incremental temporal performance over the structured predictors. That question was assessed separately in the paired analysis reported in Section 3.4.

### 3.8. Secondary Exploratory Analysis of Consultation-Response Documentation

In the secondary exploratory analysis, 300 of 656 consultations (45.7%) were assigned to an HDBSCAN cluster, whereas 356 (54.3%) were classified as Noise. Among clustered observations, 283 belonged to Cluster 1 and 17 to Cluster 2. The overall DBCV score was low (0.055), with clustering coverage of 0.457, providing limited evidence for a strongly separated and broadly distributed cluster structure (Supplementary Table S5; Supplementary Figure S1).

Robustness analyses showed that cluster agreement improved after duplicate or highly similar records were removed, although detailed stability estimates are reported in Supplementary Table S5 and Supplementary Figure S1. Response texts classified as Noise were substantially shorter than those assigned to clusters (median, 18 vs. 51 words; rank-biserial r = −0.734; *p* < 0.001). Referring-department distribution and several management outcomes also differed between Noise and clustered observations. Taken together, these patterns suggest that the embedding structure was influenced by documentation characteristics as well as clinical content. The analysis was therefore interpreted as an exploratory documentation analysis and not as evidence of validated clinical phenotypes.

## 4. Discussion

The present study examined whether information available before child and adolescent psychiatry assessment could predict six subsequent documented clinical management decisions and whether these predictive patterns remained transportable across changing service conditions. Three findings are particularly relevant. First, predictive performance was strongly management-domain specific rather than uniform across consultation outcomes, with particularly weak temporal discrimination for psychosocial management and limited performance for additional assessment. Second, temporal performance varied in a later period characterized by marked changes in referral composition and in the prevalence of several management decisions. Third, although referral-note text contributed substantially within most fitted models, its addition to routinely available structured information did not establish clear incremental temporal AUPRC after correction for multiple comparisons. The study is therefore best interpreted as an assessment of temporal transportability under changing service conditions rather than as evidence of a deployable clinical decision-support model.

The heterogeneity across management domains is clinically relevant because child and adolescent psychiatry consultation-liaison services do not represent a single prediction problem. Medication management, psychosocial intervention, additional assessment, follow-up planning, safety/disposition, and coordination with medical teams reflect partly distinct clinical processes and may depend on different combinations of patient, family, referral, and service-level information. This interpretation is consistent with the substantial heterogeneity in patient populations, service models, outcomes, and service-level effects reported across pediatric mental health liaison services [2]. Hospital-based pediatric data similarly show that acute crisis presentations, self-harm, suicidality, medical complexity, and cross-system coordination may coexist within the same consultation service [18]. Treating these decisions as a single overall consultation outcome could therefore obscure clinically and operationally meaningful differences between management domains.

Medication management decision showed the most consistent pattern across the two evaluation settings. Its temporal AUPRC was numerically close to the full-cohort cross-validated estimate, although this should not be interpreted as evidence of readiness for clinical use. The target itself was broader than medication initiation and included continuation, modification, discontinuation, conditional initiation, and explicit decisions to monitor without medication. Pre-assessment referral information may therefore capture some of the clinical context associated with medication-related decision-making more consistently than it captures other domains. For safety/disposition, temporal discrimination remained appreciable, but operating characteristics at the locked threshold were more modest. One possible explanation is that crisis- or risk-related information may be more explicitly represented in referral documentation or encoded indirectly through referral source. Although this mechanism was not tested directly, the post hoc department-level analysis showed a strong and non-uniform association between referring department and safety/disposition prevalence, ranging from 0.5% in the pediatrics outpatient clinic to 54.3% in the pediatric intensive care unit (bias-corrected Cramér’s V = 0.506; Supplementary Table S4, Panel D). This pattern is consistent with the referring-department variable capturing a mixture of clinical case mix, referral pathways, and local workflow conventions. The safety/disposition model should therefore not be assumed to generalize to institutions with different referral structures or department-coding practices.

Psychosocial management showed the weakest temporal transportability. The low temporal AUPRC occurred alongside an AUROC close to chance level, indicating that lower outcome prevalence alone does not account for the weak discrimination observed in the later cohort. AUPRC is inherently sensitive to outcome prevalence [19], but psychosocial management may also depend more heavily on information not represented as dedicated structured predictors in the present models, including family and socioeconomic context, previous psychiatric care, caregiver capacity, social resources, community-service availability, discharge options, and contemporaneous service capacity. Structured triage information and standardized social determinants of health may also contain relevant information for such context-sensitive decisions. Evidence that discharge processes in pediatric liaison settings can be influenced by psychiatric bed availability, community support, and broader resource constraints provides clinical context for this interpretation [20]. The weak performance observed here should therefore be understood both as a limitation of the available predictor set and as an indication of the difficulty of representing context-dependent psychosocial decisions using a restricted set of referral-time variables.

A central implication of the temporal evaluation is that predictive performance should not be regarded as a fixed property of a model independent of the healthcare environment. The later cohort differed substantially from the development period in referring-department composition, while the prevalence of psychosocial management, follow-up/reassessment, and liaison/medical integration also changed. Such shifts alter both predictor distributions and the base rates against which prediction performance is evaluated. Temporal dataset shift is well recognized as a challenge for clinical prediction models [7], and transportability across real-world data sources has also been shown to vary in adolescent suicide prediction, with more complex machine-learning approaches not consistently outperforming regularized logistic regression [9]. The present analysis deliberately evaluated the development-period models without recalibration, and should therefore be understood as a test of unadapted transportability across interrupted historical and service-delivery periods rather than as a simple test of model aging. For future deployment-oriented work, simple intercept recalibration could be evaluated as one adaptation strategy when changes in baseline outcome prevalence contribute to calibration drift; its adequacy would need to be assessed prospectively in the target setting.

The liaison/medical integration findings further illustrate the importance of interpreting discrimination together with outcome prevalence and service context. The prevalence of this outcome increased in the temporal cohort, accompanied by a higher AUPRC, while AUROC remained comparatively similar. Because precision–recall performance depends on the underlying event rate, the increase in AUPRC should not be interpreted independently of this changing prevalence. The effects of harmonizing the referring-department variable were similarly domain-specific. Harmonization improved some performance measures while worsening others, indicating that its principal value was not universal performance enhancement. Rather, it provided a clinically interpretable representation of referral source while reducing institution-specific administrative granularity.

The findings for referral-note text also require a distinction between within-model contribution and incremental predictive value. NLP applications in youth mental health have frequently focused on particular conditions or risk states, while documentation heterogeneity and limited generalizability remain challenges in psychiatric clinical text [21]. In the present study, referral-note text represented the largest predictive-contribution block in most SHAP analyses, yet adding text to the structured model did not produce a statistically significant improvement in temporal AUPRC after multiple-comparison correction. The relatively wide confidence intervals mean that modest incremental benefit or harm cannot be excluded. These findings are not contradictory: SHAP describes how a fitted model uses available predictors, whereas the paired structured-versus-combined comparison addressed whether text improved temporal predictive performance beyond structured information [22]. The supervised text representation should also be considered when interpreting this result. Word-level TF-IDF captures sparse lexical patterns but does not directly model broader contextual semantics or use a dedicated negation mechanism. Accordingly, the absence of established incremental temporal value in this study should not be generalized to all forms of clinical NLP. Contextual or domain-adapted language representations could be compared prospectively in future transportability studies. The BGE-M3 embeddings used in the secondary exploratory branch do not constitute such a supervised comparison because they were applied to consultation-response documentation for a separate unsupervised analysis.

The secondary unsupervised analysis provided limited evidence for a stable, broadly distributed cluster structure. More than half of consultation responses were classified as Noise, one cluster was very small, and overall DBCV was low. The association between cluster assignment and documentation length further suggests that documentation characteristics contributed to the observed embedding structure. These findings support retaining this analysis as an exploratory description of documentation structure rather than interpreting the clusters as clinical phenotypes.

### 4.1. Clinical and Service Implications

The present findings do not support automated treatment selection or autonomous clinical decision-making. Temporal performance was heterogeneous across management domains, psychosocial management showed weak discrimination and calibration, operating characteristics at fixed thresholds were limited for several outcomes, and the models have not been validated outside the study institution. Acceptable discrimination at the population level does not in itself establish useful performance at a clinical decision threshold. The more immediate implication is methodological and service-oriented. Information already available before psychiatric assessment contains measurable signal about some subsequent documented management decisions, but the usefulness of that signal depends on both the decision domain and the surrounding service context. Temporal and contextual validation are therefore prerequisites for any future attempt to incorporate routinely collected referral information into consultation workflows.

Following independent multicenter and preferably prospective validation, similar approaches could be evaluated as one component of a human-in-the-loop clinical decision-support system rather than as a substitute for psychiatric assessment or clinical judgment. Potential applications for future study could include prioritizing referrals for earlier or more intensive review, supporting organization of consultation queues, or examining whether aggregated predictions contribute to service-demand planning. Any prediction would need to be integrated with information acquired during subsequent clinical assessment rather than used to determine management autonomously. Although focused specifically on ADHD, recent work on AI-assisted clinical decision-support systems in child and adolescent mental health similarly emphasizes clinician-facing design, usability, and workflow integration as central implementation considerations [23]. The present study does not establish that any of these applications improve efficiency, resource allocation, or patient outcomes; such benefits would need prospective evaluation.

Predictive accuracy alone is therefore insufficient for implementation. Calibration, threshold-specific performance, net benefit, clinician acceptance, usability, workflow integration, and effects on clinical and service outcomes would all require evaluation. Future implementation studies should also determine whether performance remains stable across institutions with different referral practices, documentation cultures, service structures, and patient populations.

### 4.2. Strengths and Limitations

Several features strengthen the present study. Predictors were restricted to information available before psychiatric assessment, while the consultation-response text from which the outcomes were derived was excluded from the supervised models. This temporal separation reduced the risk of target leakage. Grouped resampling further prevented identical normalized referral notes from being distributed across training and evaluation partitions. The six management domains were modeled separately rather than collapsed into a single consultation outcome, allowing domain-specific differences in discrimination, calibration, and transportability to remain visible. Temporal evaluation applied models developed in the earlier period to a later service period without retraining or recalibration, providing a more demanding assessment of unadapted transportability than internal resampling alone. In addition, the high agreement obtained when 150 consultations were independently and blindly recoded by a second child and adolescent psychiatrist supports the reproducibility of the documentation-derived reference standard; period-stratified sensitivity analyses showed similarly high reproducibility in both service periods, although no formal between-period equality test was performed.

Several limitations should also be considered. First, this was a retrospective single-center study. External validity therefore remains unknown for institutions with different referral pathways, service organization, clinical populations, and documentation practices. External validation would need to examine two related but distinct forms of transportability: that of the structured predictors, particularly locally defined referring-department categories, and that of the Turkish-language referral-note features and documentation conventions. Geographic transportability and linguistic/documentation transportability should therefore not be assumed from the present temporal validation. Second, the management outcomes were derived from consultation-response documentation. They represent documented management decisions rather than a complete reconstruction of real-time clinical reasoning or an independent measure of clinical need. A score of zero within a domain indicates that the corresponding management decision was not documented according to the coding framework; it does not establish that the underlying clinical need was absent.

Clinician identifiers were unavailable after de-identification, preventing assessment of clinician-level clustering and changes in clinician composition across periods. Unmeasured clinician-level variation may therefore have contributed to heterogeneity in documented management decisions. The substantial reorganization of service delivery over time provided a useful stress test of temporal transportability, but it also means that a pure temporal effect cannot be separated from changes in referral pathways, case mix, clinician composition, and service organization.

Event counts were limited for several outcomes and exploratory subgroups, increasing uncertainty around some estimates. A related precision limitation applies to the incremental-value comparisons in Section 3.4: several ΔAUPRC 95% confidence intervals were wide relative to the point estimate, including +0.084 [+0.001, +0.154] for safety/disposition and −0.061 [−0.151, +0.012] for psychosocial management. The *n* = 209 temporal cohort therefore provides limited precision for estimating small-to-moderate incremental effects. Non-significant findings after FDR correction should consequently be read as inconclusive regarding a possible incremental contribution of referral-note text rather than as evidence that no such contribution exists. The subgroup analyses should likewise be interpreted as exploratory evidence of performance heterogeneity rather than formal assessments of algorithmic fairness or absence of bias.

The predictor set was also intentionally restricted. Family and socioeconomic context, previous psychiatric care, caregiver capacity, structured social determinants of health, structured triage information, community-service availability, and real-time service capacity were not represented as dedicated structured predictors. Their absence may be particularly consequential for context-sensitive decisions such as psychosocial management. In addition, the primary supervised text representation used word-level TF-IDF rather than contextual or domain-adapted language representations. TF-IDF offers a transparent lexical representation but may not adequately capture contextual meaning, long-range semantic relationships, or clinically relevant negation. Future work should determine whether richer pre-assessment variables and contextual language representations improve transportability rather than only internal model fit.

The explainability and unsupervised analyses have additional limitations. SHAP values describe predictive contribution within fitted models and should not be interpreted as causal effects or as evidence of independent incremental value. The HDBSCAN solution had limited coverage, low DBCV, and sensitivity to documentation characteristics and should not be interpreted as clinical phenotype discovery. Finally, the study was not prospectively preregistered, limiting the extent to which the distinction between confirmatory and exploratory analyses can be independently verified as having been specified in advance.

## 5. Conclusions

Information available before child and adolescent psychiatry assessment contained management-domain-specific predictive signal for subsequent documented clinical management decisions, but this signal was not uniformly transportable to a later service period. Temporal performance was particularly weak for psychosocial management and remained limited for additional assessment, while other domains retained varying degrees of discrimination. These findings should be interpreted alongside substantial contemporaneous changes in referral composition and outcome prevalence. Referral-note text contributed substantially within fitted models, but its addition to structured information did not yield a statistically significant incremental improvement in temporal AUPRC after correction for multiple comparisons. Given the width of the corresponding confidence intervals, this result should be regarded as inconclusive rather than as evidence that no incremental textual value exists.

These findings support interpreting the present study as an assessment of temporal transportability under changing service conditions rather than as validation of a deployable clinical decision-support model. Prediction models derived from routinely collected clinical data should be evaluated within the healthcare contexts in which they are expected to operate rather than assuming that internally observed performance will persist over time. The results support further study of data-informed consultation workflows, but not automated clinical decision-making. Multicenter external validation, followed by prospective evaluation of calibration, clinical utility, workflow integration, and effects on service and patient outcomes, is required before implementation.

## Figures and Tables

**Table 1:** Internal and temporal evaluation of the final harmonized prediction models.

Management Domain	Int. Positive *n*/*N* (%)	Int. AUPRC [95% CI]	Int. AUROC [95% CI]	Int. Brier	Temp. Positive *n*/*N* (%)	Temp. AUPRC [95% CI]	Temp. AUROC [95% CI]	Temp. Brier
Medication management decision	309/656 (47.1%)	0.662 [0.610, 0.713]	0.670 [0.629, 0.709]	0.226	101/209 (48.3%)	0.663 [0.566, 0.764]	0.664 [0.587, 0.734]	0.233
Psychosocial management	132/656 (20.1%)	0.282 [0.228, 0.360]	0.585 [0.530, 0.638]	0.159	22/209 (10.5%)	0.136 [0.079, 0.262]	0.532 [0.396, 0.665]	0.117
Additional assessment	101/656 (15.4%)	0.300 [0.225, 0.394]	0.660 [0.603, 0.715]	0.123	40/209 (19.1%)	0.282 [0.188, 0.434]	0.587 [0.481, 0.685]	0.156
Follow-up/reassessment	402/656 (61.3%)	0.721 [0.675, 0.775]	0.671 [0.626, 0.713]	0.205	96/209 (45.9%)	0.586 [0.492, 0.695]	0.649 [0.576, 0.722]	0.254
Safety/disposition	99/656 (15.1%)	0.623 [0.520, 0.722]	0.901 [0.871, 0.930]	0.083	26/209 (12.4%)	0.415 [0.248, 0.604]	0.781 [0.678, 0.873]	0.097
Liaison/medical integration	146/656 (22.3%)	0.478 [0.399, 0.568]	0.747 [0.700, 0.791]	0.147	65/209 (31.1%)	0.575 [0.464, 0.717]	0.774 [0.702, 0.845]	0.175

Note. Internal evaluation used 5-fold × 5-repeat grouped cross-validation in the full primary cohort (*n* = 656). For temporal evaluation, models were developed using 2013–2019 records (*n* = 447) and evaluated in 2022–2025 records (*n* = 209) without retraining or recalibration. AUPRC was the primary performance metric and should be interpreted alongside the corresponding outcome prevalence shown in the positive *n*/*N* (%) columns. Internal cross-validation and temporal evaluation address complementary questions and should not be treated as independent test samples. Confidence intervals were based on 2000 nonparametric bootstrap resamples. Predictors were referral-note text, age, sex, and the clinically harmonized referring department. AUPRC = area under the precision–recall curve (average precision); AUROC = area under the receiver operating characteristic curve; CI = confidence interval.

**Table 2:** Temporal operating characteristics at Youden thresholds locked in the development period.

Management Domain	Locked Youden Threshold	Sensitivity	Specificity	PPV	NPV	Balanced Accuracy
Medication management decision	0.421	0.733	0.407	0.536	0.620	0.570
Psychosocial management	0.363	0.227	0.898	0.208	0.908	0.563
Additional assessment	0.158	0.350	0.704	0.219	0.821	0.527
Follow-up/reassessment	0.715	0.375	0.770	0.581	0.592	0.572
Safety/disposition	0.272	0.577	0.885	0.417	0.936	0.731
Liaison/medical integration	0.178	0.800	0.542	0.441	0.857	0.671

Note. Thresholds were derived exclusively from grouped out-of-fold predictions in the 2013–2019 development period and applied unchanged to the 2022–2025 temporal cohort. These operating characteristics are descriptive and should not be interpreted as recommended thresholds for clinical use. PPV = positive predictive value; NPV = negative predictive value.

## Data Availability

Patient-level clinical data supporting the findings of this study are not publicly available because they include sensitive clinical information and free-text records and are subject to privacy and ethical restrictions. Reproducibility materials—including the analysis code, frozen coding guide, data dictionary, model specifications, aggregate analysis outputs, manuscript-aligned tables and figures, and synthetic/example data—are openly available in Zenodo (Version 1.0.0) at https://doi.org/10.5281/zenodo.22251191 [24]. The Zenodo record contains no patient-level clinical records or directly identifying information.

## Supplementary Materials

The following supporting information can be downloaded at: https://www.mdpi.com/article/10.3390/healthcare14213575/s1, Supplementary Table S1: Reference-standard construction, cohort derivation, outcome distributions, and inter-rater reliability; Supplementary Table S2: Final model specification, target-leakage audit, and referring-department harmonization; Supplementary Table S3: Model robustness and sensitivity analyses; Supplementary Table S4: Context of temporal transportability and exploratory subgroup performance; Supplementary Table S5: Model explainability and exploratory documentation analysis of consultation-response text; Supplementary Figure S1: Exploratory documentation structure of consultation-response text and robustness analyses; Supplementary Figure S2: Temporal calibration and exploratory decision-curve analyses; and Supplementary Figure S3: Robustness to referring-department harmonization and exploratory subgroup performance.
