# Supplementary material for: Predicting Clinical Management Decisions from Referral Information in Child and Adolescent Psychiatry Consultations: A Temporal Transportability Study
Source: Healthcare (Basel). 2026 Oct 22;14(21):3575. doi: 10.3390/healthcare14213575 (PMC13763104; doi:10.3390/healthcare14213575)
Supplement: Supplementary file 1 [file healthcare-14-03575-s001.zip › Healthcare_Supplementary_Tables_REVISED.pdf]

Supplementary Tables

Supplementary Table S1. Reference-standard construction, cohort derivation, outcome distributions, and inter-rater reliability.

Panel A. Six-domain clinical management coding framework.

Domain	Score	Operational definition (documented management decision)
Medication management decision	0	No documented medication or treatment-management decision.
Medication management decision	1	Continuation of treatment, planned/conditional initiation, medication-free follow-up, or an explicit treatment plan.
Medication management decision	2	Medication initiation/addition, dose modification, discontinuation, temporary interruption, restart, or active perioperative medication modification.
Psychosocial management	0	No documented psychosocial intervention.
Psychosocial management	1	Psychoeducation, family guidance, behavioral advice, sleep-hygiene advice, or a comparable intervention.
Psychosocial management	2	Formal psychotherapy, behavioral therapy, talking therapy, special education, or a comparable structured intervention.
Additional assessment	0	No documented additional assessment or testing.
Additional assessment	1	Additional medical/differential assessment or complementary investigation.
Additional assessment	2	Formal test or instrument (e.g., WISC/IQ, AGTE, CNSVS, teacher form, developmental test).
Follow-up/reassessment	0	No documented follow-up, reassessment, or repeat consultation.
Follow-up/reassessment	1	Routine follow-up, reassessment, or repeat consultation.
Follow-up/reassessment	2	Close reassessment within ≤7 days.
Safety/disposition	0	No documented safety/disposition decision.
Safety/disposition	1	Safety precautions, risk counseling, or an explicit low-risk assessment.
Safety/disposition	2	Admission/referral, an explicit discharge decision, or a suicide safety plan/contract.
Liaison/medical integration	0	No documented liaison/medical integration.
Liaison/medical integration	1	Evaluation by another specialty explicitly linked to exclusion of an organic cause or to the treatment plan.
Liaison/medical integration	2	Direct coordination of medical or perioperative treatment.

Panel B. Cohort derivation.

Stage/category	n	Definition
Cleaned consultation records	940	Records entering clinical validation.
Clinically usable responses	658	Consultation responses judged to contain sufficient information for clinical coding.
Research/technical-only records	110	Technical records related to a research/study process rather than a clinical consultation response.
Did not attend/could not be evaluated	95	The patient did not attend or could not be clinically evaluated.
Insufficient/brief response	55	The consultation response was too brief or insufficient for reliable clinical coding.
Test/technical-procedure-only record	22	The record contained only a test or technical procedure.
Age >18 years	2	Otherwise usable records excluded from the pediatric cohort.
Primary pediatric cohort	656	Unique patients aged 1–18 years included in supervised analyses.

Panel C. Distribution of the six management outcomes.

Management domain	Score 0	Score 1	Score 2	Overall cohort ≥1	2013–2019 ≥1	2022–2025 ≥1
Medication management decision	347	168	141	309 (47.1%)	208/447 (46.5%)	101/209 (48.3%)
Psychosocial management	524	87	45	132 (20.1%)	110/447 (24.6%)	22/209 (10.5%)
Additional assessment	555	46	55	101 (15.4%)	61/447 (13.6%)	40/209 (19.1%)
Follow-up/reassessment	254	340	62	402 (61.3%)	306/447 (68.5%)	96/209 (45.9%)
Safety/disposition	557	32	67	99 (15.1%)	73/447 (16.3%)	26/209 (12.4%)
Liaison/medical integration	510	41	105	146 (22.3%)	81/447 (18.1%)	65/209 (31.1%)

Panel D. Inter-rater reliability in the independent blinded sample.

Management domain	n	Exact 0/1/2 agreement n (%)	Linear-weighted κ [95% CI]	Quadratic-weighted κ	Gwet AC2	≥1 vs. 0 agreement n (%)	Cohen κ [95% CI]	Gwet AC1
Medication management decision	150	139 (92.7%)	0.892 [0.821, 0.951]	0.901	0.900	142 (94.7%)	0.893 [0.814, 0.960]	0.893
Psychosocial management	150	146 (97.3%)	0.919 [0.822, 0.988]	0.913	0.966	146 (97.3%)	0.921 [0.835, 0.982]	0.960
Additional assessment	150	150 (100.0%)	1.000 [1.000, 1.000]	1.000	1.000	150 (100.0%)	1.000 [1.000, 1.000]	1.000
Follow-up/reassessment	150	150 (100.0%)	1.000 [1.000, 1.000]	1.000	1.000	150 (100.0%)	1.000 [1.000, 1.000]	1.000
Safety/disposition	150	150 (100.0%)	1.000 [1.000, 1.000]	1.000	1.000	150 (100.0%)	1.000 [1.000, 1.000]	1.000
Liaison/medical integration	150	150 (100.0%)	1.000 [1.000, 1.000]	1.000	1.000	150 (100.0%)	1.000 [1.000, 1.000]	1.000

Note. A second child and adolescent psychiatrist independently re-rated a simple random sample of 150 consultations while blinded to the original ratings, referral note, model predictions, and prior results. The reliability exercise assessed reproducibility of the prespecified documentation-based coding rules rather than independent agreement on clinical need or treatment appropriateness. The sample was randomly selected and was not chosen on the basis of documentation clarity. Confidence intervals for κ coefficients were based on 5,000 paired bootstrap resamples. CI = confidence interval.

Panel E. Period-stratified inter-rater reliability of the documentation-derived reference standard.

Period	Management domain	n	R1/R2 positive n	Binary agreement n (%)	Cohen κ [95% CI]	Gwet AC1 [95% CI]	Ordinal exact agreement n (%)	Linear-weighted κ [95% CI]	Gwet AC2 [95% CI]
2013–2019	Medication management decision	92	45/48	87/92 (94.6%)	0.891 [0.783, 0.978]	0.891 [0.786, 0.978]	84/92 (91.3%)	0.885 [0.796, 0.958]	0.895 [0.818, 0.964]
2013–2019	Psychosocial management	92	21/24	89/92 (96.7%)	0.912 [0.801, 1.000]	0.948 [0.877, 1.000]	89/92 (96.7%)	0.919 [0.804, 1.000]	0.962 [0.909, 1.000]
2013–2019	Additional assessment	92	16/16	92/92 (100.0%)	1.000 [1.000, 1.000]	1.000 [1.000, 1.000]	92/92 (100.0%)	1.000 [1.000, 1.000]	1.000 [1.000, 1.000]
2013–2019	Follow-up/reassessment	92	64/64	92/92 (100.0%)	1.000 [1.000, 1.000]	1.000 [1.000, 1.000]	92/92 (100.0%)	1.000 [1.000, 1.000]	1.000 [1.000, 1.000]
2013–2019	Safety/disposition	92	19/19	92/92 (100.0%)	1.000 [1.000, 1.000]	1.000 [1.000, 1.000]	92/92 (100.0%)	1.000 [1.000, 1.000]	1.000 [1.000, 1.000]
2013–2019	Liaison/medical integration	92	19/19	92/92 (100.0%)	1.000 [1.000, 1.000]	1.000 [1.000, 1.000]	92/92 (100.0%)	1.000 [1.000, 1.000]	1.000 [1.000, 1.000]
2022–2025	Medication management decision	58	28/29	55/58 (94.8%)	0.897 [0.759, 1.000]	0.897 [0.767, 1.000]	55/58 (94.8%)	0.902 [0.775, 1.000]	0.908 [0.795, 1.000]
2022–2025	Psychosocial management	58	9/10	57/58 (98.3%)	0.937 [0.772, 1.000]	0.976 [0.920, 1.000]	57/58 (98.3%)	0.917 [0.697, 1.000]	0.972 [0.911, 1.000]
2022–2025	Additional assessment	58	11/11	58/58 (100.0%)	1.000 [1.000, 1.000]	1.000 [1.000, 1.000]	58/58 (100.0%)	1.000 [1.000, 1.000]	1.000 [1.000, 1.000]
2022–2025	Follow-up/reassessment	58	32/32	58/58 (100.0%)	1.000 [1.000, 1.000]	1.000 [1.000, 1.000]	58/58 (100.0%)	1.000 [1.000, 1.000]	1.000 [1.000, 1.000]
2022–2025	Safety/disposition	58	12/12	58/58 (100.0%)	1.000 [1.000, 1.000]	1.000 [1.000, 1.000]	58/58 (100.0%)	1.000 [1.000, 1.000]	1.000 [1.000, 1.000]
2022–2025	Liaison/medical integration	58	18/18	58/58 (100.0%)	1.000 [1.000, 1.000]	1.000 [1.000, 1.000]	58/58 (100.0%)	1.000 [1.000, 1.000]	1.000 [1.000, 1.000]

Note. The blinded reliability sample comprised 92 consultations from 2013–2019 and 58 from 2022–2025. R1/R2 positive n gives the number of positive ratings for the primary and second raters, respectively, for the binary ≥1 versus 0 outcome. Ninety-five percent confidence intervals were obtained from 5,000 paired bootstrap resamples within each period. No formal between-period comparison of reliability coefficients was performed. Complete agreement in a stratum is reported descriptively and should not be interpreted as proof of identical reliability across periods. These estimates quantify reproducibility of the documentation-derived reference standard, not independent clinical need. CI = confidence interval.

Supplementary Table S2. Final model specification, target-leakage audit, and referring-department harmonization.

Panel A. Final supervised-modeling specification.

Component	Final specification
Prediction targets	Six separate binary documentation-derived outcomes: score ≥1 vs. 0 for medication management decision, psychosocial management, additional assessment, follow-up/reassessment, safety/disposition, and liaison/medical integration. The binary target represents presence versus absence of a documented management decision, not independent clinical need.
Predictors	Referral-note text, age, sex, and the clinically harmonized referring department (17 categories). No additional structured variables representing family or socioeconomic context, previous psychiatric care, caregiver capacity, or contemporaneous service availability were included.
Excluded from the predictor set	Psychiatric consultation response text, manually coded outcomes, post-consultation information, and diagnostic fields whose pre-consultation origin could not be reliably verified.
Text normalization	Unicode NFKC normalization; normalization of Turkish dotted/dotless I; lowercasing; preservation of alphanumeric characters and Turkish letters; whitespace normalization.
Text representation	Word-level 1–2-gram TF-IDF; min_df = 3; max_df = 0.98; max_features = 12,000; sublinear term frequency; L2 normalization. Contextual embeddings, transformer-derived representations, and dedicated negation modeling were not used in the primary supervised models.
Structured predictors	Age was standardized; sex and the harmonized referring department were one-hot encoded. No predictor values were missing in the final cohort.
Classifier	L2-regularized logistic regression; C = 1.0; liblinear solver; maximum 4,000 iterations.
Internal evaluation	5-fold × 5-repeat StratifiedGroupKFold; identical normalized referral notes were retained within the same group.

Component	Final specification
Temporal evaluation	Development using 2013–2019 records (n = 447); locked evaluation in 2022–2025 records (n = 209) without retraining, recalibration, threshold optimization, or hyperparameter tuning. This evaluated unadapted temporal transportability.
Primary metric	AUPRC (average precision) was the primary metric and was interpreted alongside the corresponding outcome prevalence; AUROC and Brier score were supportive metrics.
Uncertainty	2,000 nonparametric bootstrap resamples at the consultation level; percentile 95% confidence intervals.
Random seed	20260827.
Incremental text analysis	Structured-only and combined models were trained on the same development data and evaluated on the same temporal cases. Paired ΔAUPRC quantified the incremental temporal value of referral-note text.
SHAP interpretation	SHAP quantified within-model predictive contribution only and was not used as evidence of causal effects or independent incremental temporal value.

Panel B. Duplicate-record and target-leakage audit.

Audit indicator	Value
Primary cohort, n	656
Unique normalized referral notes	644
Cases in exact-duplicate groups	22
Exact-duplicate groups	10
Largest duplicate-group size	3
Referral-response text similarity ≥0.90	0
Maximum referral-response text similarity	0.493

Note. Exactly identical normalized referral notes were retained within the same group during resampling to prevent training–test overlap. Consultation-response text, from which outcome labels were derived, was not used as a predictor in any supervised model.

Panel C. Construction of the final harmonized referring-department categories.

Final harmonized referring-department category	Primary cohort n	De-identified source-unit scope	Harmonization approach	Rationale
Pediatrics outpatient clinic	201	Pediatrics outpatient units, including numbered and after-hours administrative variants	Administrative label normalization	Equivalent outpatient labels were merged while preserving specialty and care setting.
Pediatrics inpatient service	104	Pediatrics inpatient service	Retained as distinct category	Retained because the specialty and inpatient care setting were clinically distinct.
Pediatric intensive care unit	92	Pediatric intensive care service	Retained as distinct category	Retained because the intensive care setting was clinically distinct.
Anesthesiology outpatient clinic	39	Anesthesiology outpatient units, including numbered administrative variants	Administrative label normalization	Equivalent anesthesiology outpatient labels were merged.
Pediatric emergency department	36	Pediatric emergency service	Retained as distinct category	Retained because the emergency care setting was clinically distinct.
Other surgical outpatient clinics	32	Low-frequency surgical outpatient services, including general surgery, urology, neurosurgery, orthopedics, plastic surgery, and oral/maxillofacial surgery	Low-frequency clinical grouping	Low-frequency surgical outpatient services were grouped while preserving outpatient care setting.
Orthopedics inpatient service	22	Orthopedics inpatient service	Retained as distinct category	Retained because the specialty and inpatient care setting were clinically distinct.
Other outpatient clinics	20	Low-frequency non-surgical outpatient services, including ophthalmology, neurology, psychiatry, rehabilitation, and pediatric neurology	Low-frequency clinical grouping	Low-frequency non-surgical outpatient services were grouped while preserving outpatient care setting.
Otorhinolaryngology outpatient clinic	18	Otorhinolaryngology outpatient units, including numbered administrative variants	Administrative label normalization	Equivalent otorhinolaryngology outpatient labels were merged.
Other surgical inpatient services	17	Low-frequency surgical inpatient services, including general surgery, neurosurgery, pediatric surgery, and oral/maxillofacial surgery	Low-frequency clinical grouping	Low-frequency surgical inpatient services were grouped while preserving inpatient care setting.
Other intensive care units	13	Low-frequency non-pediatric intensive care services, including neurosurgical, orthopedic, emergency, and internal-medicine intensive care	Low-frequency clinical grouping	Low-frequency intensive care services were grouped while preserving the intensive care setting.
Plastic and reconstructive surgery service	13	Plastic and reconstructive surgery inpatient service	Retained as distinct category	Retained because the specialty and inpatient care setting were clinically distinct.
Dermatology outpatient clinic	12	Dermatology outpatient units, including numbered and after-hours administrative variants	Administrative label normalization	Equivalent dermatology outpatient labels were merged.
Anesthesiology/maxillofacial surgery outpatient clinic	10	Combined perioperative anesthesiology and maxillofacial-surgery outpatient service	Retained as distinct combined category	Retained to preserve the specific perioperative referral context.
Physical medicine and rehabilitation service	10	Physical medicine and rehabilitation inpatient service	Retained as distinct category	Retained because the specialty and inpatient care setting were clinically distinct.
Pediatric surgery outpatient clinic	10	Pediatric surgery outpatient service	Retained as distinct category	Retained because the specialty and outpatient care setting were clinically distinct.
Other inpatient services	7	Low-frequency non-surgical inpatient services, including internal medicine, neurology, obstetrics/gynecology, dermatology, and ophthalmology	Low-frequency clinical grouping	Low-frequency non-surgical inpatient services were grouped while preserving inpatient care setting.

Note. To preserve institutional and personnel privacy, raw administrative labels are not reproduced. The table summarizes the de-identified unit types contributing to each final category and the harmonization rule applied. Administrative numbering and after-hours variants were collapsed when clinical specialty and care setting were unchanged. Low-frequency units were grouped only into clinically coherent higher-level categories while preserving care setting where possible. The resulting 17-category variable was used as the harmonized referring-department predictor in the primary models.

Supplementary Table S3. Model robustness and sensitivity analyses.

Panel A. Paired sensitivity analysis of raw versus harmonized referring-department coding.

Evaluation setting	Management domain	Metric	Raw	Harmonized	Δ (harmonized − raw)	95% CI	p	BH-FDR q
5×5 repeated grouped CV	Medication management decision	AUPRC	0.662	0.662	+0.001	[-0.016, +0.019]	0.936	0.958
5×5 repeated grouped CV	Medication management decision	AUROC	0.674	0.670	-0.005	[-0.019, +0.010]	0.523	0.628
5×5 repeated grouped CV	Medication management decision	Brier	0.226	0.226	-0.000	[-0.005, +0.004]	0.998	0.998
5×5 repeated grouped CV	Psychosocial management	AUPRC	0.291	0.282	-0.009	[-0.022, +0.003]	0.125	0.255
5×5 repeated grouped CV	Psychosocial management	AUROC	0.601	0.585	-0.016	[-0.031, -0.002]	0.026	0.078
5×5 repeated grouped CV	Psychosocial management	Brier	0.158	0.159	+0.002	[+0.000, +0.003]	0.046	0.092
5×5 repeated grouped CV	Additional assessment	AUPRC	0.315	0.300	-0.016	[-0.039, +0.006]	0.170	0.255
5×5 repeated grouped CV	Additional assessment	AUROC	0.658	0.660	+0.002	[-0.019, +0.023]	0.893	0.893
5×5 repeated grouped CV	Additional assessment	Brier	0.122	0.123	+0.001	[-0.001, +0.003]	0.485	0.728
5×5 repeated grouped CV	Follow-up/reassessment	AUPRC	0.739	0.721	-0.018	[-0.043, +0.006]	0.137	0.255
5×5 repeated grouped CV	Follow-up/reassessment	AUROC	0.694	0.671	-0.023	[-0.045, -0.000]	0.048	0.096
5×5 repeated grouped CV	Follow-up/reassessment	Brier	0.200	0.205	+0.005	[+0.000, +0.009]	0.043	0.092
5×5 repeated grouped CV	Safety/disposition	AUPRC	0.639	0.623	-0.016	[-0.034, -0.000]	0.045	0.255
5×5 repeated grouped CV	Safety/disposition	AUROC	0.904	0.901	-0.003	[-0.008, +0.002]	0.255	0.383
5×5 repeated grouped CV	Safety/disposition	Brier	0.081	0.083	+0.002	[+0.000, +0.003]	0.020	0.092
5×5 repeated grouped CV	Liaison/medical integration	AUPRC	0.477	0.478	+0.001	[-0.025, +0.024]	0.958	0.958
5×5 repeated grouped CV	Liaison/medical integration	AUROC	0.729	0.747	+0.017	[+0.007, +0.029]	<0.001	<0.001
5×5 repeated grouped CV	Liaison/medical integration	Brier	0.148	0.147	-0.001	[-0.003, +0.002]	0.644	0.773
Temporal 2013–2019 → 2022–2025	Medication management decision	AUPRC	0.653	0.663	+0.010	[-0.029, +0.052]	0.600	0.720
Temporal 2013–2019 → 2022–2025	Medication management decision	AUROC	0.649	0.664	+0.015	[-0.009, +0.042]	0.245	0.367
Temporal 2013–2019 → 2022–2025	Medication management decision	Brier	0.235	0.233	-0.002	[-0.008, +0.004]	0.541	0.910
Temporal 2013–2019 → 2022–2025	Psychosocial management	AUPRC	0.144	0.136	-0.008	[-0.050, +0.010]	0.411	0.659
Temporal 2013–2019 → 2022–2025	Psychosocial management	AUROC	0.529	0.532	+0.003	[-0.035, +0.043]	0.964	0.964
Temporal 2013–2019 → 2022–2025	Psychosocial management	Brier	0.117	0.117	+0.000	[-0.003, +0.003]	0.922	0.922
Temporal 2013–2019 → 2022–2025	Additional assessment	AUPRC	0.321	0.282	-0.039	[-0.066, -0.007]	0.003	0.018
Temporal 2013–2019 → 2022–2025	Additional assessment	AUROC	0.612	0.587	-0.024	[-0.050, -0.002]	0.034	0.068
Temporal 2013–2019 → 2022–2025	Additional assessment	Brier	0.153	0.156	+0.003	[+0.001, +0.005]	0.004	0.024

Evaluation setting	Management domain	Metric	Raw	Harmonized	Δ (harmonized − raw)	95% CI	p	BH-FDR q
Temporal 2013–2019 → 2022–2025	Follow-up/reassessment	AUPRC	0.584	0.586	+0.002	[-0.049, +0.050]	0.989	0.989
Temporal 2013–2019 → 2022–2025	Follow-up/reassessment	AUROC	0.639	0.649	+0.010	[-0.023, +0.044]	0.551	0.661
Temporal 2013–2019 → 2022–2025	Follow-up/reassessment	Brier	0.255	0.254	-0.001	[-0.011, +0.008]	0.767	0.920
Temporal 2013–2019 → 2022–2025	Safety/disposition	AUPRC	0.429	0.415	-0.014	[-0.057, +0.012]	0.439	0.659
Temporal 2013–2019 → 2022–2025	Safety/disposition	AUROC	0.762	0.781	+0.019	[+0.006, +0.037]	0.003	0.009
Temporal 2013–2019 → 2022–2025	Safety/disposition	Brier	0.096	0.097	+0.001	[-0.002, +0.003]	0.607	0.910
Temporal 2013–2019 → 2022–2025	Liaison/medical integration	AUPRC	0.544	0.575	+0.031	[-0.022, +0.083]	0.240	0.659
Temporal 2013–2019 → 2022–2025	Liaison/medical integration	AUROC	0.733	0.774	+0.042	[+0.018, +0.068]	<0.001	<0.001
Temporal 2013–2019 → 2022–2025	Liaison/medical integration	Brier	0.187	0.175	-0.012	[-0.023, -0.002]	0.011	0.033

Note. Δ values were calculated from unrounded estimates and then rounded; therefore, subtraction of the displayed raw and harmonized values may differ from Δ by ±0.001 in some rows. Positive Δ values for AUPRC/AUROC and negative Δ values for Brier score favor harmonization. Multiple comparisons were adjusted within each metric/evaluation family using the Benjamini–Hochberg false discovery rate (BH-FDR) procedure. CI = confidence interval; CV = cross-validation.

Panel B. Sensitivity to L2 regularization strength.

Management domain	C	Internal AUPRC	Internal AUROC	Internal Brier	Temporal AUPRC	Temporal AUROC	Temporal Brier
Medication management decision	0.1	0.652	0.659	0.230	0.590	0.607	0.242
Medication management decision	0.3	0.663	0.669	0.226	0.639	0.644	0.237
Medication management decision	1	0.662	0.670	0.226	0.663	0.664	0.233
Medication management decision	3	0.647	0.661	0.235	0.658	0.654	0.244
Medication management decision	10	0.624	0.645	0.254	0.620	0.626	0.277
Psychosocial management	0.1	0.316	0.591	0.156	0.206	0.512	0.117
Psychosocial management	0.3	0.308	0.597	0.156	0.165	0.515	0.114
Psychosocial management	1	0.282	0.585	0.159	0.136	0.532	0.117
Psychosocial management	3	0.252	0.573	0.167	0.131	0.549	0.131
Psychosocial management	10	0.232	0.564	0.181	0.128	0.558	0.160
Additional assessment	0.1	0.255	0.633	0.127	0.221	0.565	0.157
Additional assessment	0.3	0.286	0.650	0.124	0.244	0.581	0.157
Additional assessment	1	0.300	0.660	0.123	0.282	0.587	0.156

Management domain	C	Internal AUPRC	Internal AUROC	Internal Brier	Temporal AUPRC	Temporal AUROC	Temporal Brier
Additional assessment	3	0.296	0.664	0.124	0.313	0.582	0.156
Additional assessment	10	0.290	0.659	0.129	0.315	0.570	0.161
Follow-up/reassessment	0.1	0.691	0.647	0.213	0.561	0.601	0.279
Follow-up/reassessment	0.3	0.703	0.662	0.206	0.592	0.641	0.268
Follow-up/reassessment	1	0.721	0.671	0.205	0.586	0.649	0.254
Follow-up/reassessment	3	0.727	0.672	0.210	0.568	0.642	0.257
Follow-up/reassessment	10	0.725	0.669	0.224	0.556	0.633	0.279
Safety/disposition	0.1	0.540	0.883	0.094	0.367	0.760	0.102
Safety/disposition	0.3	0.584	0.892	0.088	0.384	0.765	0.099
Safety/disposition	1	0.623	0.901	0.083	0.415	0.781	0.097
Safety/disposition	3	0.636	0.903	0.081	0.447	0.788	0.097
Safety/disposition	10	0.616	0.898	0.084	0.424	0.782	0.102
Liaison/medical integration	0.1	0.466	0.745	0.149	0.573	0.768	0.195
Liaison/medical integration	0.3	0.476	0.750	0.146	0.578	0.774	0.184
Liaison/medical integration	1	0.478	0.747	0.147	0.575	0.774	0.175
Liaison/medical integration	3	0.462	0.734	0.153	0.567	0.768	0.180
Liaison/medical integration	10	0.440	0.712	0.164	0.555	0.756	0.203

Note. C = 1.0 was the locked primary specification and was not re-selected after the sensitivity analysis.

Panel C. Sensitivity analysis for high-intensity management decisions (score 2 vs. 0–1).

Management domain	Score 2, n	Prevalence	AUPRC [95% CI]	AUROC	Brier	Calibration slope
Medication management decision	141	0.215	0.518 [0.436, 0.608]	0.760	0.139	0.784
Psychosocial management	45	0.069	0.071 [0.050, 0.103]	0.526	0.069	0.079
Additional assessment	55	0.084	0.221 [0.139, 0.333]	0.737	0.073	0.724
Follow-up/reassessment	62	0.095	0.214 [0.156, 0.300]	0.739	0.081	0.714
Safety/disposition	67	0.102	0.619 [0.498, 0.745]	0.923	0.053	1.035
Liaison/medical integration	105	0.160	0.417 [0.331, 0.520]	0.794	0.113	0.768

Note. This panel is an archived development-stage supportive analysis assessing whether the overall performance pattern depended materially on combining lower- and higher-intensity documented management decisions in the primary ≥1 versus 0 definition. It was not used to change the primary outcome definition or select the final model. CI = confidence interval.

Panel D. Incremental temporal AUPRC of referral text beyond structured predictors.

Management domain	ΔAUPRC (text + structured − structured only)	95% CI	BH-FDR q
Medication management decision	+0.013	[-0.044, +0.074]	0.653
Psychosocial management	-0.061	[-0.151, +0.012]	0.576
Additional assessment	+0.031	[-0.030, +0.106]	0.576
Follow-up/reassessment	+0.016	[-0.053, +0.089]	0.653
Safety/disposition	+0.084	[+0.001, +0.154]	0.288

Management domain	ΔAUPRC (text + structured − structured only)	95% CI	BH-FDR q
Liaison/medical integration	+0.017	[-0.038, +0.092]	0.653

Note. Combined and structured-only models were trained on the same 2013–2019 development data and compared in the same 2022–2025 temporal cases. Confidence intervals were based on 2,000 paired bootstrap resamples; q values are Benjamini–Hochberg FDR-adjusted across the six management domains. Positive values favor the combined model. Non-significant q values are interpreted together with the confidence intervals and should not be taken as evidence that incremental textual signal is absent. Values correspond to final Figure 2D. CI = confidence interval.

Supplementary Table S4. Context of temporal transportability and exploratory subgroup performance.

Panel A. Change in referring-department service mix.

Harmonized referring department	2013–2019 n (%)	2022–2025 n (%)	Change (percentage points)	Temporal status
Pediatrics outpatient clinic	155 (34.7%)	46 (22.0%)	-12.7	Present in both periods
Pediatrics inpatient service	67 (15.0%)	37 (17.7%)	+2.7	Present in both periods
Pediatric intensive care unit	75 (16.8%)	17 (8.1%)	-8.6	Present in both periods
Anesthesiology outpatient clinic	2 (0.4%)	37 (17.7%)	+17.3	Present in both periods
Pediatric emergency department	22 (4.9%)	14 (6.7%)	+1.8	Present in both periods
Other surgical outpatient clinics	29 (6.5%)	3 (1.4%)	-5.1	Present in both periods
Orthopedics inpatient service	10 (2.2%)	12 (5.7%)	+3.5	Present in both periods
Other outpatient clinics	19 (4.3%)	1 (0.5%)	-3.8	Present in both periods
Otorhinolaryngology outpatient clinic	18 (4.0%)	0 (0.0%)	-4.0	Absent from temporal cohort
Other surgical inpatient services	8 (1.8%)	9 (4.3%)	+2.5	Present in both periods
Other intensive care units	12 (2.7%)	1 (0.5%)	-2.2	Present in both periods
Plastic and reconstructive surgery service	8 (1.8%)	5 (2.4%)	+0.6	Present in both periods
Dermatology outpatient clinic	4 (0.9%)	8 (3.8%)	+2.9	Present in both periods
Anesthesiology/maxillofacial surgery outpatient clinic	0 (0.0%)	10 (4.8%)	+4.8	Not observed in development period
Physical medicine and rehabilitation service	4 (0.9%)	6 (2.9%)	+2.0	Present in both periods
Pediatric surgery outpatient clinic	10 (2.2%)	0 (0.0%)	-2.2	Absent from temporal cohort
Other inpatient services	4 (0.9%)	3 (1.4%)	+0.5	Present in both periods

Panel B. Change in outcome prevalence.

Management domain	Development events/n (%)	Temporal events/n (%)	Risk difference (temporal − development)	95% CI	BH-FDR q
Medication management decision	208/447 (46.5%)	101/209 (48.3%)	+1.8 pp	[-6.3, +9.9]	0.668
Psychosocial management	110/447 (24.6%)	22/209 (10.5%)	-14.1 pp	[-19.5, -7.9]	<0.001
Additional assessment	61/447 (13.6%)	40/209 (19.1%)	+5.5 pp	[-0.4, +12.0]	0.104
Follow-up/reassessment	306/447 (68.5%)	96/209 (45.9%)	-22.5 pp	[-30.3, -14.4]	<0.001
Safety/disposition	73/447 (16.3%)	26/209 (12.4%)	-3.9 pp	[-9.2, +2.2]	0.234
Liaison/medical integration	81/447 (18.1%)	65/209 (31.1%)	+13.0 pp	[+6.0, +20.3]	<0.001

Panel C. Exploratory subgroup performance in the temporal cohort.

Management domain	Subgroup variable	Group	Positive n/N (%)	AUPRC [95% CI]	AUROC [95% CI]	Brier	Sensitivity	Specificity
Medication management decision	Sex	Male	57/99 (57.6%)	0.770 [0.653, 0.880]	0.729 [0.622, 0.823]	0.211	0.860	0.381
Medication management decision	Sex	Female	44/110 (40.0%)	0.465 [0.337, 0.639]	0.548 [0.436, 0.658]	0.252	0.568	0.424

Management domain	Subgroup variable	Group	Positive n/N (%)	AUPRC [95% CI]	AUROC [95% CI]	Brier	Sensitivity	Specificity
Medication management decision	Age group	1–11 years	41/83 (49.4%)	0.741 [0.602, 0.875]	0.744 [0.634, 0.845]	0.209	0.732	0.476
Medication management decision	Age group	12–18 years	60/126 (47.6%)	0.632 [0.503, 0.746]	0.609 [0.505, 0.702]	0.248	0.733	0.364
Psychosocial management	Sex	Male	10/99 (10.1%)	0.142 [0.059, 0.359]	0.506 [0.274, 0.729]	0.121	0.300	0.865
Psychosocial management	Sex	Female	12/110 (10.9%)	0.150 [0.072, 0.332]	0.561 [0.367, 0.739]	0.113	0.167	0.929
Psychosocial management	Age group	1–11 years	8/83 (9.6%)*	0.184 [0.083, 0.429]	0.672 [0.449, 0.877]	0.129	0.625	0.760
Psychosocial management	Age group	12–18 years	14/126 (11.1%)	0.120 [0.070, 0.224]	0.504 [0.342, 0.672]	0.109	0.000	0.991
Additional assessment	Sex	Male	20/99 (20.2%)	0.323 [0.187, 0.556]	0.622 [0.473, 0.765]	0.158	0.550	0.570
Additional assessment	Sex	Female	20/110 (18.2%)	0.263 [0.143, 0.437]	0.564 [0.415, 0.708]	0.153	0.150	0.822
Additional assessment	Age group	1–11 years	19/83 (22.9%)	0.305 [0.170, 0.517]	0.498 [0.317, 0.668]	0.181	0.579	0.328
Additional assessment	Age group	12–18 years	21/126 (16.7%)	0.352 [0.191, 0.558]	0.634 [0.488, 0.771]	0.139	0.143	0.933
Follow-up/reassessment	Sex	Male	33/99 (33.3%)	0.534 [0.395, 0.710]	0.731 [0.628, 0.829]	0.258	0.424	0.803
Follow-up/reassessment	Sex	Female	63/110 (57.3%)	0.638 [0.523, 0.766]	0.560 [0.452, 0.666]	0.249	0.349	0.723
Follow-up/reassessment	Age group	1–11 years	24/83 (28.9%)	0.510 [0.351, 0.710]	0.739 [0.624, 0.843]	0.267	0.375	0.864
Follow-up/reassessment	Age group	12–18 years	72/126 (57.1%)	0.642 [0.537, 0.767]	0.584 [0.486, 0.686]	0.245	0.375	0.667
Safety/disposition	Sex	Male	8/99 (8.1%)*	0.204 [0.073, 0.524]	0.716 [0.540, 0.879]	0.076	0.250	0.934
Safety/disposition	Sex	Female	18/110 (16.4%)	0.503 [0.304, 0.725]	0.804 [0.678, 0.909]	0.116	0.722	0.837
Safety/disposition	Age group	1–11 years	5/83 (6.0%)*	0.205 [0.068, 0.515]	0.818 [0.638, 0.951]	0.057	0.000	1.000
Safety/disposition	Age group	12–18 years	21/126 (16.7%)	0.471 [0.284, 0.688]	0.782 [0.669, 0.889]	0.123	0.714	0.800
Liaison/medical integration	Sex	Male	45/99 (45.5%)	0.648 [0.510, 0.814]	0.771 [0.674, 0.867]	0.208	0.911	0.389
Liaison/medical integration	Sex	Female	20/110 (18.2%)	0.331 [0.182, 0.531]	0.659 [0.515, 0.787]	0.144	0.550	0.633
Liaison/medical integration	Age group	1–11 years	33/83 (39.8%)	0.662 [0.503, 0.835]	0.772 [0.670, 0.869]	0.197	0.818	0.600
Liaison/medical integration	Age group	12–18 years	32/126 (25.4%)	0.521 [0.370, 0.725]	0.765 [0.659, 0.858]	0.160	0.781	0.511

*Note. The distribution of referring departments differed between periods ($\chi^2(16) = 159.62$, $p < 0.001$; Cramér's V = 0.493). Subgroup analyses were post hoc, descriptive performance audits rather than prespecified tests of between-group differences or algorithmic fairness. * Fewer than 10 positive events; estimates should be interpreted with particular caution. CI = confidence interval.*

Panel D. Safety/disposition prevalence by harmonized referring-department category and period.

Harmonized referring department	Full n	Full safety/disposition positive n (%) [95% CI]	2013–2019 n	2013–2019 positive n (%) [95% CI]	2022–2025 n	2022–2025 positive n (%) [95% CI]
Pediatrics outpatient clinic	201	1 (0.5%) [0.1%, 2.8%]	155	1 (0.6%) [0.1%, 3.6%]	46	0 (0.0%) [0.0%, 7.7%]
Pediatrics inpatient service	104	15 (14.4%) [8.9%, 22.4%]	67	6 (9.0%) [4.2%, 18.2%]	37	9 (24.3%) [13.4%, 40.1%]
Pediatric intensive care unit	92	50 (54.3%) [44.2%, 64.1%]	75	43 (57.3%) [46.1%, 67.9%]	17	7 (41.2%) [21.6%, 64.0%]
Anesthesiology outpatient clinic	39	0 (0.0%) [0.0%, 9.0%]	2	0 (0.0%) [0.0%, 65.8%]	37	0 (0.0%) [0.0%, 9.4%]
Pediatric emergency department	36	11 (30.6%) [18.0%, 46.9%]	22	9 (40.9%) [23.3%, 61.3%]	14	2 (14.3%) [4.0%, 39.9%]
Other surgical outpatient clinics	32	0 (0.0%) [0.0%, 10.7%]	29	0 (0.0%) [0.0%, 11.7%]	3	0 (0.0%) [0.0%, 56.1%]
Orthopedics inpatient service	22	5 (22.7%) [10.1%, 43.4%]	10	1 (10.0%) [1.8%, 40.4%]	12	4 (33.3%) [13.8%, 60.9%]
Other outpatient clinics	20	7 (35.0%) [18.1%, 56.7%]	19	7 (36.8%) [19.1%, 59.0%]	1	0 (0.0%) [0.0%, 79.3%]
Otorhinolaryngology outpatient clinic	18	0 (0.0%) [0.0%, 17.6%]	18	0 (0.0%) [0.0%, 17.6%]	0	0 / not estimable
Other surgical inpatient services	17	3 (17.6%) [6.2%, 41.0%]	8	1 (12.5%) [2.2%, 47.1%]	9	2 (22.2%) [6.3%, 54.7%]
Plastic and reconstructive surgery service	13	0 (0.0%) [0.0%, 22.8%]	8	0 (0.0%) [0.0%, 32.4%]	5	0 (0.0%) [0.0%, 43.4%]
Other intensive care units	13	3 (23.1%) [8.2%, 50.3%]	12	3 (25.0%) [8.9%, 53.2%]	1	0 (0.0%) [0.0%, 79.3%]
Dermatology outpatient clinic	12	0 (0.0%) [0.0%, 24.2%]	4	0 (0.0%) [0.0%, 49.0%]	8	0 (0.0%) [0.0%, 32.4%]
Pediatric surgery outpatient clinic	10	0 (0.0%) [0.0%, 27.8%]	10	0 (0.0%) [0.0%, 27.8%]	0	0 / not estimable
Physical medicine and rehabilitation service	10	2 (20.0%) [5.7%, 51.0%]	4	0 (0.0%) [0.0%, 49.0%]	6	2 (33.3%) [9.7%, 70.0%]
Anesthesiology/maxillofacial surgery outpatient clinic	10	0 (0.0%) [0.0%, 27.8%]	0	0 / not estimable	10	0 (0.0%) [0.0%, 27.8%]
Other inpatient services	7	2 (28.6%) [8.2%, 64.1%]	4	2 (50.0%) [15.0%, 85.0%]	3	0 (0.0%) [0.0%, 56.1%]

Note. Wilson 95% confidence intervals are shown for department-specific prevalence. Empty period-specific categories were retained to preserve the 17-category model structure. The association between harmonized referring department and safety/disposition was substantial in the full cohort ($\chi^2(16) = 183.73$; Monte Carlo $p < 0.001$; bias-corrected Cramér's V = 0.506), remained pronounced in 2013–2019 ($\chi^2(15) = 157.43$; Monte Carlo $p < 0.001$; bias-corrected V = 0.565), and was still evident in 2022–2025 ($\chi^2(14) = 41.93$; Monte Carlo $p = 0.0007$; bias-corrected V = 0.366). Because several expected cell counts were <5, inference was based primarily on a fixed-margin Monte Carlo conditional test with 50,000 replicates rather than the asymptotic chi-square p value.

Supplementary Table S5. Model explainability and exploratory documentation analysis of consultation-response text.

Panel A. Predictor-block contribution in the final harmonized models.

Management domain	Referral-note text	Harmonized referring department	Age	Sex
Medication management decision	57.4%	27.3%	8.5%	6.7%
Psychosocial management	50.6%	20.4%	10.8%	18.1%
Additional assessment	47.3%	19.0%	12.3%	21.4%
Follow-up/reassessment	66.4%	29.1%	0.4%	4.1%
Safety/disposition	30.9%	35.4%	16.6%	17.0%
Liaison/medical integration	44.6%	39.0%	4.7%	11.7%

Panel B. Exploratory HDBSCAN solution and robustness indicators.

Analysis	Cluster/assignment	n/repeats	Proportion	Metric	Value	Additional metric
Cluster assignment	Noise (density-based non-assignment)	356	0.543	HDBSCAN membership probability	0.000	—
Cluster assignment	Cluster 1	283	0.431	HDBSCAN membership probability	0.835	—

Analysis	Cluster/assignment	n/repeats	Proportion	Metric	Value	Additional metric
Cluster assignment	Cluster 2	17	0.026	HDBSCAN membership probability	0.997	—
Cluster assignment	Total clustered	300	0.457	—	—	—
Subsample stability	Cluster 1	25	—	Matched Jaccard	0.890	p10 = 0.846
Subsample stability	Cluster 2	25	—	Matched Jaccard	0.750	p10 = 0.489
Full-text vs. detemplated agreement	All cases	656	—	AMI	0.544	ARI = 0.599; matched Jaccard = 0.763
Sensitivity to removal of exact/near-duplicate records	All cases	646	—	AMI	0.848	ARI = 0.910; matched Jaccard = 0.947

Note. The values 0.835 and 0.997 in the cluster-assignment rows are HDBSCAN membership probabilities, not cluster persistence. Coverage = 0.457 and overall DBCV = 0.055. The moderate agreement between full-text and detemplated solutions indicates sensitivity of the semantic structure to documentation templates and argues against interpretation as validated clinical phenotype discovery. AMI = adjusted mutual information; ARI = adjusted Rand index; DBCV = density-based clustering validation.

Panel C. Descriptive characteristics of Noise and clustered cases.

Group	n	%	Age, median (IQR)	Referral word count, median (IQR)	Response word count, median (IQR)	Female, %	Age 12–18 years, %	Medication management ≥1	Psychosocial management ≥1	Additional assessment ≥1	Follow-up ≥1	Safety ≥1	Liaison ≥1
Clustered	300	45.7%	14 (10–16)	14 (9–21)	51 (38–73)	54.7%	66.0%	51.3%	22.3%	17.3%	72.0%	23.0%	27.0%
Noise (density-based non-assignment)	356	54.3%	11 (6–15)	13 (8–18)	18 (11–26)	51.4%	48.6%	43.5%	18.3%	13.8%	52.2%	8.4%	18.3%

Panel D. Statistical comparisons of Noise and clustered cases.

Variable	Test	Effect size	Effect-size metric	p	BH-FDR q*
Age	Mann–Whitney U	-0.234	Rank-biserial r (Noise − Clustered)	<0.001	—
Referral-note character count	Mann–Whitney U	-0.084	Rank-biserial r (Noise − Clustered)	0.063	—
Referral-note word count	Mann–Whitney U	-0.095	Rank-biserial r (Noise − Clustered)	0.035	—
Response-text character count	Mann–Whitney U	-0.724	Rank-biserial r (Noise − Clustered)	<0.001	—
Response-text word count	Mann–Whitney U	-0.734	Rank-biserial r (Noise − Clustered)	<0.001	—
Template-removal fraction	Mann–Whitney U	-0.034	Rank-biserial r (Noise − Clustered)	0.131	—
Female sex	Chi-square	-0.033	Risk difference (Noise − Clustered)	0.404	—
12–18 years	Chi-square	-0.174	Risk difference (Noise − Clustered)	<0.001	—
2022–2025 period	Chi-square	0.047	Risk difference (Noise − Clustered)	0.202	—
Medication management decision	Chi-square	-0.078	Risk difference (Noise − Clustered)	0.046	0.070
Psychosocial management	Chi-square	-0.041	Risk difference (Noise − Clustered)	0.195	0.207
Additional assessment	Chi-square	-0.036	Risk difference (Noise − Clustered)	0.207	0.207
Follow-up/reassessment	Chi-square	-0.198	Risk difference (Noise − Clustered)	<0.001	<0.001
Safety/disposition	Chi-square	-0.146	Risk difference (Noise − Clustered)	<0.001	<0.001

Variable	Test	Effect size	Effect-size metric	p	BH-FDR q*
Liaison/medical integration	Chi-square	-0.087	Risk difference (Noise − Clustered)	0.007	0.015
Harmonized referring department	Chi-square	0.182	Bias-corrected Cramér V	0.002	—

*Note. SHAP values are presented as shares of total mean absolute SHAP contribution and describe predictive contribution within fitted models. They do not establish causal effects, treatment effects, clinical decision rules, or independent incremental temporal value beyond the structured predictors. "Noise" denotes density-based non-assignment in the embedding space and does not indicate a clinically unclassifiable patient. The six management outcomes were not used as clustering inputs. * BH-FDR q values were calculated for the six management-outcome comparisons. SHAP = SHapley Additive exPlanations; BH-FDR = Benjamini–Hochberg false discovery rate.*
